# Taurine Attenuates Disuse Muscle Atrophy Through Modulation of the xCT-GSH-GPX4 and AMPK-ACC-ACSL4 Pathways

**DOI:** 10.3390/antiox14070847

**Published:** 2025-07-10

**Authors:** Xi Liu, Yifen Chen, Linglin Zhang, Zhen Qi, Longhe Yang, Caihua Huang, Li Wang, Donghai Lin

**Affiliations:** 1 Key Laboratory for Chemical Biology of Fujian Province, College of Chemistry and Chemical Engineering, Xiamen University, Xiamen 361005, China; 2 Technical Innovation Center for Utilization of Marine Biological Resources, Third Institute of Oceanography, Ministry of Natural Resources, Xiamen 361005, China; longheyang@tio.org.cn; 3 Research and Communication Center of Exercise and Health, Xiamen University of Technology, Xiamen 361024, China; huangcaihua@xmut.edu.cn; 4 School of Nursing, Suzhou Medical College of Soochow University, Suzhou 215123, China; li-wang-1@suda.edu.cn

**Keywords:** taurine, disuse muscle atrophy, ferroptosis, xCT-GSH-GPX4 signaling, AMPK-ACC-ACSL4 pathway, skeletal muscle metabolism

## Abstract

Disused muscle atrophy (DMA) is characterized by skeletal muscle loss and functional decline due to prolonged inactivity. Though evidence remains limited, recent studies suggest that ferroptosis, an iron-dependent, lipid peroxidation-driven form of cell death, may contribute to DMA. Taurine, a natural amino acid enriched in energy drinks, can improve the proliferation and myogenic differentiation potential of myoblasts. This study aimed to investigate whether taurine supplementation could protect against DMA and explore its potential role in modulating ferroptosis. Using a hindlimb suspension-induced DMA model in male C57BL/6J mice (6–8 weeks old), we assessed muscle mass, function, ferroptosis-related markers, histopathological changes, and metabolic alterations. The results showed that taurine supplementation improved muscle strength and morphology while attenuating markers of ferroptosis, including iron accumulation, lipid peroxidation, and glutathione and related protein (NRF2, GPX4, and xCT) depletion. Metabolomic analysis suggested that taurine modulates disorders in glutathione and lipid metabolism, potentially associated with the regulation of the xCT-GSH-GPX4 and AMPK-ACC-ACSL4 pathways. While these findings support a protective role for taurine and a possible link between ferroptosis and DMA, further functional studies are needed to confirm causality and assess the compound’s translational potential. This study provides initial in vivo evidence implicating ferroptosis in DMA and highlights taurine as a promising candidate for future therapeutic exploration.

## 1. Introduction

Skeletal muscle, the largest organ in the human body, plays a vital role in maintaining posture, physical activity, thermogenesis, and metabolic regulation [1]. Skeletal muscle atrophy, characterized by a significant loss of muscle mass and a decrease in myofiber cross-sectional area, can result from genetic mutations, aging, immobilization or disuse, cancer, and other pathological conditions [2,3,4,5]. In particular, disuse muscle atrophy (DMA) results from extended inactivity and is associated with multiple cellular stress responses, including oxidative stress, mitochondrial dysfunction, inflammation, impaired autophagy, and apoptosis [6,7]. Apoptosis has long been recognized as a significant contributor to DMA, with studies reporting the activation of caspase-3 and mitochondrial-mediated apoptotic signaling in skeletal muscle following hindlimb unloading or immobilization [8,9]. However, apoptosis alone does not fully account for the underlying mechanisms of DMA, and emerging evidence suggests that alternative forms of regulated cell death, particularly ferroptosis, may play an underappreciated role in DMA pathogenesis [10,11].

Ferroptosis, characterized by iron-dependent lipid peroxidation, has recently been implicated in various muscle-wasting conditions associated with aging, cisplatin treatment, and sepsis [10]. The xCT-glutathione (GSH)-GPX4 axis plays a central role in antioxidant defense, mediating cystine uptake for GSH synthesis and enabling GPX4 to detoxify lipid peroxides [12,13,14]. Concurrently, the AMPK-ACC-ACSL4 pathway regulates lipid metabolism and ferroptosis sensitivity by modulating the availability of polyunsaturated phospholipids (PUFA-PLs), which are susceptible to peroxidation [15,16]. While both pathways are well characterized in other biological contexts, their combined role in the progression of DMA has not been fully elucidated. Investigating these pathways together may reveal whether ferroptosis contributes to skeletal muscle degradation via dual impairments in redox balance and lipid metabolic regulation, thereby addressing a critical gap in current knowledge.

Taurine, a naturally occurring β-sulfonic acid, is abundant in excitable tissues and is commonly found in energy drinks, fish, and meat [17]. It has multiple biological functions including antioxidant and anti-inflammatory effects, and it has been shown to attenuate skeletal muscle atrophy [18,19,20,21]. Our previous studies have shown that taurine alleviates cisplatin-induced myotube atrophy and ferroptosis-impaired myoblasts [22,23], suggesting a potential novel mechanism for its protective effects in muscle disorders. However, the mechanistic effects of taurine on ferroptosis and associated metabolic pathways in the context of DMA remain unclear.

We thus hypothesize that taurine alleviates DMA by inhibiting ferroptosis through regulation of the xCT-GSH-GPX4 and AMPK-ACC-ACSL4 signaling pathways. Using in vivo hindlimb suspension and in vitro C2C12 myotube models, combined with NMR-based metabolomic analysis and molecular assays, this study aims to elucidate the modulatory effects of taurine and provide mechanistic insight into targeting ferroptosis for muscle preservation.

## 2. Materials and Methods

### 2.1. Reagents and Instruments

The following reagents and equipment were used in this study: taurine (T0625, Sigma-Aldrich, Shanghai, China), erastin (HY-15763, MedChemExpress, Shanghai, China), methanol (80080418, AR, SinoPharm, Beijing, China), chloroform (10006818, AR, SinoPharm, Beijing, China), and D_2_O and TSP (Qingdao Tenglong Microwave Technology Co., Ltd., Qingdao, China). Assay kits included MDA (BC0025, Solarbio, Beijing, China), iron assay kit (A039-2-1, Nanjing Jiancheng, Nanjing, China), BCA (LabLead), ROS (E004-1-1, Nanjing Jiancheng Bioengineering Institute, Nanjing, China), GSH (A006-2-1, Nanjing Jiancheng Bioengineering Institute, Nanjing, China), γ-GCS (A091-1-1, Nanjing Jiancheng Bioengineering Institute, Nanjing, China), and GPx (S0058, Beyotime, Shanghai, China). Additional materials and equipment included FerroOrange (F374, Dojindo, Shanghai, China), an ultrapure water system (Milli-Q, Darmstadt, Germany), a fluorescence microscope (Motic, Xiamen, China), an ultra-high-resolution confocal laser microscope (Leica, Wetzlar, Germany), a Bruker Avance III HD 850 MHz spectrometer (Bruker, München, Germany), a multimode microplate reader (BioTek, Winooski, VT, USA), and a freeze-dryer (LGJ-10E, Foring Technology Development, Beijing Co., Ltd., Beijing, China).

### 2.2. Data Reporting

The chosen sample sizes were similar to those used in the field: *n*  =  9 samples were used to evaluate the levels of metabolites in tissues [24]; *n*  =  4–9 samples were used to determine the activity of GPx and γ-GCS [25,26]; *n*  =  4–9 samples were used for the determination of total iron content, MDA, and total GSH [27,28,29]; *n*  =  3 samples were used for the assessment of gastrocnemius muscle contractility [30]; and *n*  =  3 samples were used to determine the expression levels and phosphorylation levels of a specific protein [31]. No statistical methods were used to predetermine the sample size. Each experiment was designed and performed along with proper controls, and samples for comparison were collected and analyzed under the same conditions. Randomization was applied where applicable. For NMR analysis, samples were processed and analyzed in random order. In the animal study, mice were randomly allocated into four groups using computer-generated random numbers. For in vitro assays, cells were seeded and assigned to treatment groups in parallel using random assignment. Randomization was not performed in specific technical workflows (e.g., Western blot), where samples needed to be loaded in a specific order to generate the final figures. Blinding was applied wherever possible. For example, samples, cages, or cell-culture dishes during sample collection and processing were labelled as code names that were later revealed by the individual who picked and treated animals or cells but did not participate in sample collection and processing until assessing the outcome. Similarly, during microscopy data collection and statistical analyses, the fields of view were chosen on a random basis and were often performed by different operators, which prevented potentially biased selection for desired phenotypes.

### 2.3. Disused Muscle Atrophy Animal Experiment

**Figure 1:**
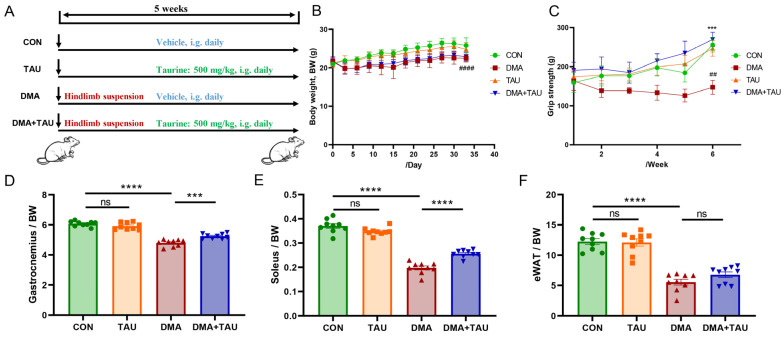
Taurine attenuates disuse muscle atrophy (DMA) in mice. (**A**) Experimental design illustrating hindlimb suspension-induced DMA and taurine intervention. (**B**) Body weight trajectory of mice over the experimental period, showing that mice in the DMA group had slower weight gain, while taurine had no significant effect on body weight (Biological replicates, *n* = 9). (**C**) Weekly grip strength measurements showing a significant reduction in grip strength after hindlimb suspension, which was restored by taurine supplementation (Biological replicates, *n* = 9). Data are expressed as mean ± SEM and analyzed using one-way ANOVA with Tukey’s post hoc test. Statistical significance: DMA vs. CON group, ^##^ *p* < 0.01, ^####^ *p* < 0.0001; DMA + TAU vs. DMA group, *** *p* < 0.001. (**D**–**F**) Quantification of skeletal muscle and fat indices (biological replicates, *n* = 9): (**D**) gastrocnemius index (gastrocnemius weight/body weight), (**E**) soleus index (soleus weight/body weight), (**F**) epididymal fat index (epididymal fat weight/body weight), indicating reduced indexes after hindlimb suspension, which was alleviated by taurine. Data are expressed as mean ± SEM and analyzed using one-way ANOVA with Tukey’s post hoc test, with statistical significance: ns for *p* > 0.05, *** *p* < 0.001, **** *p* < 0.0001.

Male C57BL/6J mice (6–8 weeks old, 20–22 g) were housed in a controlled environment (12 h light/dark cycle, 70 ± 5% relative humidity, 23 ± 2 °C). Following a 3–5-day acclimatization period, the mice were randomly assigned to four groups (*n* = 9 per group) according to generated random numbers: Control (CON), Taurine-Controlled (TAU), Disused Muscle Atrophy Model (DMA), and Taurine-Treated (DMA + TAU). A schematic of the study design is shown in Figure 1A. The protocol of these animal experiments adhered to the guidelines outlined by the China Council on Animal Care and Use and was approved by the Institutional Animal Care and the Animal Committee of Xiamen University (XMULAC20220299).

The disused muscle atrophy animal model was established under the hindlimb suspension procedure described in previous studies [32,33]. Briefly, the mice’s tails were carefully secured using medical-grade cotton threads and suspended at an appropriate height to prevent hindlimb contact with the ground, simulating a microgravity-induced unloading condition. Food and water were freely accessible throughout the experiment.

Taurine (500 mg/kg, T0625, Sigma) was administered intragastrically once daily to the TAU and DMA + TAU groups throughout the experimental period. The selected dosage was based on prior studies that demonstrated its efficacy in preserving skeletal muscle integrity under pathological conditions [34]. Body weight and grip strength were recorded at regular intervals.

After five weeks, a comprehensive evaluation of skeletal muscle strength and functional performance was conducted as described in subsequent sections. Gastrocnemius samples were collected for metabolomics, molecular biology, and histopathological analyses.

### 2.4. Grip Strength Test

Grip strength was tested using a Grip Strength Measuring (GSM) instrument (47200, Ugo Basile (Varese, Italy)). We placed each mouse on the grid of the instrument so that it naturally gripped the grid. Then, we slowly and horizontally pulled the mouse’s tail backwards until the mouse released the grid. The maximum grip force was recorded, and the test was repeated three times after the mouse had rested sufficiently.

### 2.5. Assessment of Gastrocnemius Muscle Contractility

The gastrocnemius muscle was carefully dissected and immediately immersed in a Petri dish containing Krebs solution. One end of the muscle was secured to a precision transducer using surgical sutures, while the other was attached to a fixed hook at the base of a chamber filled with a specially formulated physiological saline solution designed to mimic the natural ionic environment of skeletal muscle. This solution contained 10 mM glucose, 2.5 mM CaCl_2_, 10 mM HEPES buffer, 140 mM NaCl, 5 mM KCl, and 2 mM MgCl_2_.

The contractility assessment procedure was completed within three minutes, including preparation steps such as exposing the Achilles tendon, gastrocnemius, and soleus muscles. Electrodes were attached to the gastrocnemius muscle to initiate contraction testing. The maximum contractile force (T_max_) (*n* = 3) was recorded using the BL-420F Biosignal Acquisition and Analysis System (v2.4.0.179, Chengdu Taimeng Software Co., Ltd., Chengdu, China). Muscle fatigue resistance (*n* = 3) was evaluated based on the time required for T_max_ to decrease to half of its initial value (1/2 T_max_) [35].

Following the assessments, humane euthanasia was performed via cervical dislocation. The gastrocnemius muscle was immediately dissected and snap-frozen in liquid nitrogen to ensure protocol uniformity and procedural consistency across all experimental groups.

### 2.6. Histopathology and Immunohistochemistry

The gastrocnemius muscle samples were fixed in 4% paraformaldehyde for 24 h, followed by graded ethanol dehydration and embedding in a paraffin matrix for sectioning into 5 μm thick slices. The sections were stained with hematoxylin and eosin (H&E), and high-resolution images were captured using a light microscope equipped with digital imaging technology. The cross-sectional area of individual muscle fibers was measured using ImageJ software (version 1.51j8), and data visualization was performed using GraphPad Prism.

For immunohistochemistry, the 5 μm thick paraffin sections were deparaffinized in xylene, rehydrated through graded ethanol solutions (100%, 95%, 85%, and 75%), and subjected to antigen retrieval via microwave heating in citrate buffer. Endogenous peroxidase activity was blocked with 3% H_2_O_2_ in methanol, followed by serum blocking to reduce nonspecific binding. The sections were then incubated overnight at 4 °C with a COX2-specific primary antibody, followed by incubation with HRP-conjugated goat anti-rat IgG for 1 h at 37 °C. The signal was developed using DAB working solution, and sections were counterstained with hematoxylin.

### 2.7. Tissue Iron Measurement

The total iron content in the gastrocnemius tissue (*n* = 4) was quantified using an iron assay kit, following the manufacturer’s protocol. Briefly, tissue samples (100 mg) were homogenized in 900 μL of normal saline using a 65 Hz grinder for 60 s at 4 °C. The homogenate was centrifuged at 10,000× *g* for 10 min, and the supernatant was mixed with a chromogenic reagent. The mixture was centrifuged again and incubated in a boiling water bath for 5 min. After cooling, the supernatant was transferred to a 96-well plate, and absorbance at 520 nm was measured using a microplate reader (BioTek, Winooski, VT, USA).

### 2.8. Cell Lines and Cell Culture

Murine C2C12 myoblasts were obtained from the National Biomedical Experimental Cell Resource Bank (Beijing, China). Cells were maintained at 37 °C in high-glucose DMEM supplemented with 10% fetal bovine serum (Biological Industries, Beit HaEmek, Israel), 100 U/mL penicillin, and 100 μg/mL streptomycin under a humidified 5% CO_2_ atmosphere. When the myoblasts reached 85–90% confluence, differentiation into myotubes was induced by switching to DMEM supplemented with 2% horse serum, 100 U/mL penicillin, and 100 μg/mL streptomycin. The differentiation medium was replaced daily until cell harvest. Taurine (5 mM) was added to the differentiation medium as required. Morphological evaluation was performed using an inverted microscope at 20× magnification.

Differentiated myotubes were assigned to three experimental groups: control (CON)—untreated myotubes; erastin-treated (ERA)—myotubes treated with 2 μM erastin; erastin + taurine (ERA + TAU)—myotubes treated with 2 μM erastin and 5 mM taurine.

### 2.9. MDA Measurement

Malondialdehyde (MDA) levels were quantified using an MDA assay kit according to the manufacturer’s protocol. Briefly, myotubes or gastrocnemius samples (*n* = 4–5) were lysed and subjected to ultrasonication (200 W, 3 s pulse-on/10 s pulse-off cycles, repeated 30 times). The lysates were centrifuged at 8000× *g* for 10 min at 4 °C, and 100 μL of the supernatant was mixed with 400 μL of thiobarbituric acid working solution. The samples were incubated in a 100 °C water bath for 60 min, cooled on ice, and centrifuged again (10,000× *g* for 10 min). The supernatant was transferred to 96-well plates, and absorbance at 532 nm and 600 nm was measured using a multimode microplate reader. Data were normalized to protein concentration to ensure accuracy.

### 2.10. Measurement of the Total GSH

The total GSH content in myotubes or gastrocnemius samples (*n* = 6–9) was measured using the total GSH kit. The extracted solution was added to the samples, followed by vortexing and homogenization. The homogenate was centrifuged at 3500 rpm for 10 min at 4 °C, and the supernatant was collected and mixed with the reactive solution for 5 min. The absorbance at 405 nm was measured to determine the total GSH content, which was subsequently quantified. Data were normalized to protein concentration to ensure accuracy.

### 2.11. Assessment of GPx Enzyme Activities

Myotubes or gastrocnemius samples (*n* = 4–9) were lysed in lysis buffer, and the supernatant was collected after centrifugation at 12,000× *g* for 10 min at 4 °C. A 50 µL aliquot of the supernatant was transferred to a 96-well plate, followed by the addition of 40 µL of a working solution containing NADPH, GSH, and glutathione reductase. The mixture was incubated at room temperature for 15 min, after which 10 µL of a peroxide reagent was added to initiate the reaction. Absorbance at 340 nm was measured immediately using a multimode microplate reader. Measurements were recorded every minute for six time points per sample to determine glutathione peroxidase (GPx) activity. The data were normalized to protein concentration to ensure accuracy.

### 2.12. Detection of Fe^2+^ in Myotubes Using Laser Scanning Confocal Microscopy

Myoblasts were seeded on glass-bottom dishes and differentiated for 10 days. Following treatment with specific reagents, myotubes were double-stained with FerroOrange (1 μM) and DAPI (100 μM) in serum-free medium for 30 min at 37 °C in a temperature-controlled incubator. After rinsing with PBS, high-resolution digital images were captured using an ultrahigh-resolution confocal laser microscope (Leica TCS SP8 STED 3X, Leica Microsystems, Wetzlar, Germany) equipped with a 63× oil objective. Image analysis was conducted using LAS X Office software (version _4.7.0, Leica Microsystems, Germany).

### 2.13. Detection of ROS

To assess intracellular ROS levels, myoblasts were seeded in 6 cm culture dishes. Following differentiation, myotubes (*n* = 3) were incubated with 5 μM H_2_DCFDA (dissolved in PBS) for 30 min at 37 °C in a temperature-controlled incubator. After incubation, myotubes were washed with PBS, and representative images from different experimental groups were captured using a fluorescence microscope (Motic, Xiamen, China). Quantitative analysis was performed using ImageJ software (National Institutes of Health, Bethesda, MD, USA) based on three representative images per group.

### 2.14. Assessment of γ-GCS Enzyme Activities

Myotubes (*n* = 5) were lysed in normal saline, and the supernatant was collected after centrifugation at 12,000× *g* for 10 min at 4 °C. A 10 µL aliquot of the supernatant was transferred to 1.5 mL tubes, followed by the addition of 110 µL of working solution. After incubation at 37 °C for 6 min, 10 µL of terminator solution was added and mixed thoroughly. Next, 400 µL of chromogenic agent and stabilizer were added.

The absorbance at 636 nm was immediately measured using a multimode microplate reader. γ-GCS enzyme activity was calculated and normalized to protein concentration to ensure accuracy.

### 2.15. Western Blotting

Proteins were extracted from myotubes and gastrocnemius muscle samples (biological replicates, *n* = 3) using RIPA lysis buffer, followed by sonication and centrifugation at 12,000× *g* for 15 min at 4 °C. Total protein concentration was determined using a BCA protein assay kit, and the supernatant was mixed with SDS loading buffer.

For immunoblotting, 10–30 μg of total protein was separated on 10% SDS-PAGE and transferred to PVDF membranes. Membranes were blocked with 5% BSA for 60 min at room temperature and incubated overnight at 4 °C with the following primary antibodies at their respective working dilutions: MuRF1 (1:1000, 55456-1-AP, Proteintech, Wuhan, China), Atrogin1 (1:1000, 67172-1-Ig, Proteintech, Wuhan, China), NRF2 (1:1000, 20733, Cell Signaling Technology, Danvers, MA, USA), SLC7A11/xCT (1:1000, 26864-1-AP, Proteintech, Wuhan, China), GPX4 (1:1000, 67763-1-Ig, Proteintech, Wuhan, China), ACSL4 (1:1000, 66617-1-Ig, Proteintech, Wuhan, China), Phospho-AMPKα (1:1000, 50081T, Cell Signaling Technology, Danvers, MA, USA), AMPKα (1:1000, 5831T, Cell Signaling Technology, Danvers, MA, USA), Phospho-ACC (1:1000, 11818T, Cell Signaling Technology, Danvers, MA, USA), ACC (1:1000, 3676T, Cell Signaling Technology, Danvers, MA, USA), and β-Actin (1:3000, 20536-1-AP, Proteintech, Wuhan, China). For immunohistochemistry, COX2 antibody was used at 1:200 dilution (HY-P80090, MedChemExpress, Shanghai, China).

After washing, the membranes were incubated with a horseradish peroxidase (HRP)-conjugated secondary antibody for 1 h at room temperature. Protein bands were visualized using an enhanced chemiluminescence (ECL) reagent and captured using ChemiScope Capture (ChemiScope 6000, CLiNX, Shanghai, China). Band intensity was analyzed with ChemiScope Analysis software (Version 7.1, CLiNX, Shanghai, China) and ImageJ (1.51j8, Bethesda, MD, USA) software. Each blot was normalized to a validated internal control (such as β-actin) to account for the loading differences.

### 2.16. NMR Sample Preparation and Spectra Acquisition

NMR experiments for metabolomics analysis were conducted as previously described [33]. Briefly, gastrocnemius tissues (biological replicates, *n* = 9) were weighed, and approximately 100 mg of each sample was subjected to a solvent mixture of methanol, chloroform, and water (4:4:2.85, *v*/*v*/*v*) to extract aqueous metabolites. Samples were homogenized using a 65 Hz grinder at 4 °C for 60 s. The extracted aqueous phase was dried under nitrogen gas and reconstituted in 550 μL of NMR buffer, consisting of 100% D_2_O, 50 mM phosphate buffer, and 0.5 mM TSP (pH 7.4).

The ^1^H-NMR spectra were acquired using a Bruker Avance III HD 850 MHz spectrometer equipped with a TCI cryoprobe (Bruker BioSpin, Ettlingen, Germany). The standard NOESYGPPR1D pulse sequence [RD-G1-90°-τm-G2-90°-ACQ] was applied to suppress water signals and enhance metabolite detection. The key experimental parameters for ^1^H-NMR spectra were as follows: mixing time (τm), 10 ms; acquisition time (ACQ), 2.66 s; relaxation delay (RD), 4 s; number of transients, 64; data points, 64 K; spectral width, 15 ppm. All spectra were acquired under optimized conditions to ensure high-resolution metabolite profiling.

The 2D ^1^H-^13^C HSQC spectrum was acquired using the hsqcetgpsisp2.2 pulse sequence (Topspin v4.0, Bruker, Ettlingen, Germany). The spectral width in the ^1^H dimension is 15 ppm, the spectral width in the ^13^C dimension is 130 ppm, the relaxation delay is 1.5 s, and the sampling data matrix has 1024 × 128 points.

### 2.17. NMR Spectra Processing

Raw NMR spectral data were preprocessed using MestReNova 9.0 software (Mestrelab Research S.L., Santiago de Compostela, Spain). Resonance assignments were performed using Chenomx NMR Suite 8.3 (Chenomx Inc., Edmonton, Canada), in conjunction with relevant literature references [32].

The water resonance (δ 4.85–4.75 ppm) was excluded, and the remaining spectral regions (δ 9.50–0.50 ppm) were segmented into 0.001 ppm bins for feature extraction. A data matrix was generated using MATLAB R2014b (MathWorks, Natick, MA, USA).

Peak integrals were normalized to the TSP integral and tissue mass to ensure consistency across samples. Relative metabolite concentrations were calculated based on characteristic peaks and proton counts and are expressed as mean ± standard error of the mean (SEM). This approach enabled accurate quantitative comparisons between experimental groups.

### 2.18. Univariate and Multivariate Statistical Analyses of NMR Data

Univariate statistical analysis was performed using one-way ANOVA, followed by post hoc tests to assess group differences. Tukey’s Honestly Significant Difference (HSD) test was applied for homoscedastic data, while the Games–Howell test was used for heteroscedastic data. All analyses were conducted using SPSS 22.0 software (IBM, New York, NY, USA). Differential metabolites were identified based on an FDR-adjusted q-value (Benjamini-Hochberg method) < 0.05, ensuring statistical significance.

Multivariate statistical analysis was performed using SIMCA-P 14.1 software (Umetrics, Umeå, Sweden), including unsupervised principal component analysis (PCA) and supervised partial least squares discriminant analysis (PLS-DA). PCA provided an unbiased visualization of sample clustering and variation, while PLS-DA was applied to model group discrimination in a supervised manner. Model reliability and robustness were assessed through 200 permutation tests (*n* = 200), where R^2^Y (cum) and Q^2^Y (cum) values close to 1 indicated a strong predictive model.

Significant metabolites were identified based on a Variable Importance in Projection (VIP) score > 1 from the PLS-DA model. Characteristic metabolites were further determined based on dual selection criteria, requiring an FDR q < 0.05 (from univariate analysis) and VIP > 1 (from PLS-DA). These metabolites were subsequently analyzed to explore their biological significance and contribution to the observed metabolic differences between groups.

### 2.19. Metabolic Pathway Analysis

The concentrations of identified metabolites across different experimental groups were uploaded to MetaboAnalyst 5.0 software for metabolic pathway analysis. Significantly altered metabolic pathways were identified based on two criteria: *p*-value < 0.05 (equivalent to −lg *p* > 1.3) from metabolite set enrichment analysis; Pathway Impact Value (PIV) > 0.2 from pathway topology analysis. This integrated approach ensured the identification of biologically meaningful and statistically significant metabolic pathways associated with group differences.

### 2.20. General Statistical Analysis

All experimental data are presented as mean ± SEM. One-way ANOVA was used for comparisons involving more than two groups, performed in IBM SPSS Statistics 22.0. For post hoc analysis, Tukey’s HSD test was applied for homoscedastic data, whereas the Games–Howell test was used for heteroscedastic data. Statistical significance was indicated as follows: * *p* < 0.05, ** *p* < 0.01, *** *p* < 0.001, **** *p* < 0.0001. Data visualization and histogram plotting were conducted using GraphPad Prism 8.0.

## 3. Results

### 3.1. Taurine Alleviates Disuse Muscle Atrophy

To investigate the protective effects of taurine on DMA, we performed animal experiments as shown in Figure 1A. Body weight records revealed that mice subjected to hindlimb suspension had slower weight gain, while taurine supplementation had no significant effect on body weight (Figure 1B). Weekly grip strength assessments revealed that hindlimb suspension led to an obvious reduced grip strength, whereas taurine administration significantly improved grip strength in DMA mice (Figure 1C). In addition, hindlimb suspension markedly decreased the index of the gastrocnemius, soleus, and epididymal fat, with taurine treatment effectively restoring indexes of skeletal muscle while with no significant effect on fat (Figure 1D–F).

**Figure 2:**
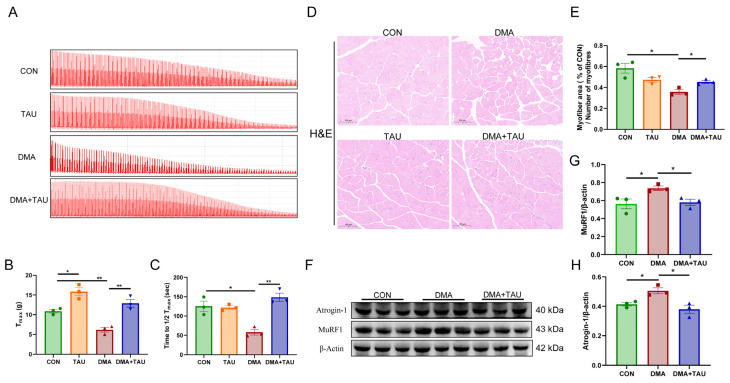
Taurine improves muscle function and alleviates histopathological changes in DMA mice. (**A**) Representative contractility curve of ex vivo gastrocnemius muscle. (**B**) Maximum contractile force (T_max_) analysis showing reduced muscle strength in DMA mice, which was significantly restored by taurine supplementation (biological replicates, *n* = 3). (**C**) Assessment of fatigue resistance, measured as the time required for T_max_ to decrease to half its initial value (1/2T_max_). Taurine intervention significantly improved fatigue resistance in DMA mice (biological replicates, *n* = 3). (**D**,**E**) Hematoxylin and eosin (H&E) staining of gastrocnemius cross sections, scale bar: 100 μm, total magnification: 200×; (**D**) quantitative comparisons of myofiber CSA between groups (biological replicates, *n* = 3); (**E**) DMA caused a significant reduction in myofiber cross-sectional area (CSA), whereas taurine treatment effectively preserved myofiber size. (**F**–**H**) Western blot analysis (**F**) and grayscale analysis (**G**,**H**) of muscle atrophy markers MuRF1 and atrogin-1 in the gastrocnemius muscle (biological replicates, *n* = 3). DMA upregulated these atrophic proteins, whereas taurine supplementation significantly decreased their expression. Data are expressed as mean ± SEM and analyzed using one-way ANOVA with Tukey’s post hoc test. Statistical significance: * *p* < 0.05, ** *p* < 0.01.

Muscle contractility and endurance were assessed based on maximal contraction force (T_max_) and fatigue resistance (time required for T_max_ to decrease to 1/2T_max_). Analysis of isolated gastrocnemius muscle showed that hindlimb suspension significantly reduced both T_max_ and 1/2T_max_, indicating impaired muscle function. However, taurine supplementation effectively improved muscle strength and endurance in DMA mice (Figure 2A–C). Histopathological analysis using H&E staining revealed that the cross-sectional area (CSA) of gastrocnemius myofibers was significantly reduced in the DMA mice compared to the controls. Notably, taurine treatment significantly increased the CSA of myofiber (Figure 2D,E).

MuRF1 and atrogin-1, two muscle-specific E3 ubiquitin ligases, are key markers of muscle atrophy that are upregulated under atrophic conditions [36]. Both were significantly elevated in the gastrocnemius of DMA mice, while taurine supplementation effectively suppressed their expression (Figure 2F–H).

### 3.2. Taurine Inhibits Ferroptosis in DMA

**Figure 3:**
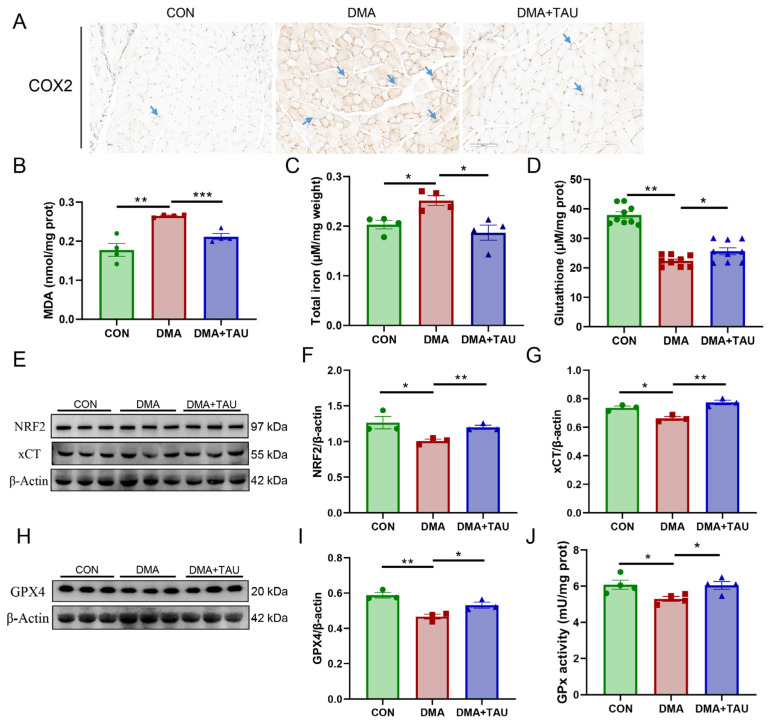
Taurine inhibits ferroptosis in the gastrocnemius muscle of DMA mice. (**A**) Immuno-histochemical staining for COX2 (blue arrows), a marker of lipid peroxidation, showing increased expression in DMA mice, which was significantly reduced by taurine treatment. Scale bar: 100 μm. Total magnification: 200×. (**B**) MDA levels, a key marker of lipid peroxidation, showing a significant increase in DMA mice and a marked reduction after taurine supplementation (biological replicates, *n* = 4). (**C**) Iron accumulation in the gastrocnemius muscle of DMA nice, a characteristic feature of ferroptosis, was significantly increased in DMA mice and attenuated by taurine treatment (biological replicates, *n* = 4). (**D**) Total GSH level was significantly decreased in the gastrocnemius muscle of DMA mice and increased by taurine treatment (biological replicates, *n* = 9). (**E**–**G**) Western blot analysis (**E**) and grayscale analysis of NRF2 (**F**) and xCT (**G**). The expression of NRF2 and xCT was significantly reduced in DMA mice, whereas taurine treatment restored their levels (biological replicates, *n* = 3). (**H**,**I**) Western blot analysis (**H**) and grayscale analysis of GPX4 (**I**), the key regulators in the ferroptosis defense system. The expression of GPX4 was significantly reduced in DMA mice, whereas taurine treatment restored their levels (biological replicates, *n* = 3). (**J**) The enzymatic activity of GPx showed that DMA resulted in decreased GPx activity, whereas taurine improved it (biological replicates, *n* = 4). Data are expressed as mean ± SEM and analyzed using one-way ANOVA with Tukey’s post hoc test. Statistical significance: * *p* < 0.05, ** *p* < 0.01, *** *p* < 0.001 vs. DMA.

To evaluate the potential involvement of ferroptosis in DMA, we first examined the expression of cyclooxygenase 2 (COX2), the catalyst of the peroxidation and a marker gene of ferroptosis [37]. Immunohistochemical staining showed significant upregulation of COX2 in DMA mice (Figure 3A), which was significantly reduced by taurine treatment.

We next assessed key biochemical indicators of ferroptosis, including MDA levels, total iron content, and total GSH levels. Compared to the controls, the DMA mice exhibited significantly increased MDA and iron accumulation, along with a marked reduction in GSH levels in the gastrocnemius muscle (Figure 3B–D). These changes were significantly attenuated by taurine supplementation.

To further investigate the ferroptosis defense system, we analyzed the expression of xCT, GPX4, and their upstream regulator, nuclear factor erythroid 2-related factor 2 (NRF2). The results showed that NRF2, xCT, and GPX4 expression were significantly decreased in DMA mice, along with a reduction in GPx enzymatic activity, confirming the impairment of the ferroptosis defense system (Figure 3E–J), strongly suggesting the presence of ferroptosis in DMA-induced muscle atrophy. And, taurine treatment partially restored NRF2, xCT, and GPX4 expression and increased GPx activity, suggesting that taurine might attenuate ferroptosis in DMA by restoring redox homeostasis.

### 3.3. Taurine Modulates Glutathione and Lipid Metabolism to Alleviate DMA

Ferroptosis is the consequence of complex perturbations in iron, amino acids, and lipid metabolism [38]. To investigate the metabolic changes underlying the regulatory effects of DMA and taurine, we performed NMR-based metabolomics analysis on gastrocnemius samples. A total of 42 metabolites were identified in the ^1^H-NMR spectra, with their resonance signals annotated in Figures S1 and S2 and Table S1. These data were normalized to TSP and subjected to multivariate and univariate statistical analyses.

**Figure 4:**
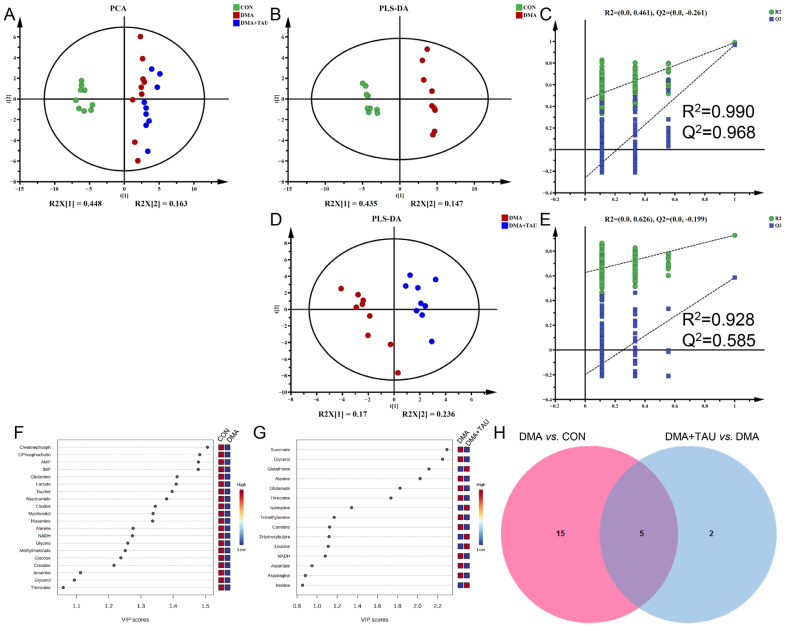
Taurine modulates metabolic disturbances in the gastrocnemius muscle of DMA mice. (**A**) PCA score plot showing distinct metabolic profiles among the three groups (CON, DMA, and DMA + TAU) (biological replicates, *n* = 9). (**B**,**D**) PLS-DA score plots for group comparisons (biological replicates, *n* = 9): (**B**) DMA vs. CON, (**D**) DMA + TAU vs. DMA, showing sharp, distinct metabolic profiles between groups. (**C**,**E**) Validation of the PLS-DA models for DMA vs. CON (**C**) and DMA + TAU vs. DMA (**E**) using 200 permutation tests, demonstrating the robustness and predictive ability of the models. (**F**,**G**) VIP score ranking of metabolites with VIP > 1 from PLS-DA models for the DMA vs. CON (**F**) and DMA + TAU vs. DMA (**G**) comparisons, highlighting significant metabolic changes. (**H**) Venn diagram illustrating the overlap of characteristic metabolites identified from the PLS-DA models of DMA vs. CON and DMA + TAU vs. DMA.

PCA and PLS-DA score plots revealed a clear separation of metabolic profiles from the control mice (CON), whereas taurine intervention resulted in a distinct metabolic signature that partially reversed the DMA-associated shifts (Figure 4A,B,D). Permutation testing (N = 200) validated the robustness of the PLS-DA models, showing that the Q^2^Y (cum) and R^2^Y (cum) values from randomized models were consistently lower than the established models, with both values converging close to 1 (Figure 4C,E). These results confirmed the strong explanatory and predictive capabilities of the established PLS-DA models, thus ensuring the validity of the metabolomics data.

Differential metabolites were screened via one-way ANOVA with Benjamini–Hochberg correction using the criterion of FDR q < 0.05 (Table S2). Significant metabolites that significantly contributed to the metabolic separation between groups were identified using the criterion of PLS-DA derived VIP > 1 (Figure 4F,G). Characteristic metabolites were identified using the criteria of FDR q < 0.05 and VIP > 1, revealing five common characteristic metabolites, glycerol, alanine, glutamine, threonine, and NADH, that were consistently altered in both the DMA vs. CON group and the DMA + TAU vs. DMA group (Figure 4H and Table S3). These results indicate widespread metabolic dysfunction in DMA, with taurine playing a role in its modulation and recovery.

Metabolic pathway analysis based on the identified metabolites revealed seven dysregulated metabolic pathways in DMA (Figure S3 and Table S4), including alanine, aspartate, and glutamate metabolism; taurine and hypotaurine metabolism; glutathione metabolism; histidine metabolism; starch and sucrose metabolism; glycine, serine, and threonine metabolism; and glycerolipid metabolism. Among these, taurine intervention significantly modulated three key pathways. These pathways involve critical metabolites such as glutamate, glutathione, NADH, choline, sn-glycero-3-phosphocholine, and glycerol, supporting the notion that taurine relieves DMA by regulating glutathione homeostasis and lipid metabolism (Table S5).

### 3.4. Taurine Regulates Glutathione and Glycerolipid Metabolism via the xCT-GSH-GPX4 and AMPK-ACC-ACSL4 Pathways in Gastrocnemius

Metabolomics analysis revealed significant alterations in several key metabolites involved in glutathione and glycerolipid metabolism. Glutathione metabolism plays a pivotal role in maintaining redox homeostasis and protecting against ferroptosis, with key enzymes such as xCT, GPX4, and GPx facilitating glutathione synthesis and utilization [12,14,39]. In DMA mice, we observed significant reductions in xCT and GPX4 protein expression, GPx enzymatic activity, and total GSH levels in gastrocnemius muscle, which are central to the xCT-GSH-GPX4 pathway, indicating impaired antioxidant defenses and increased susceptibility to ferroptosis (Figure 3D–J). Taurine intervention markedly upregulated xCT and GPX4 expression, and it increased GPx activity and GSH levels (Figure 3D–H). These findings suggest that taurine enhances glutathione metabolism via xCT-GSH-GPX4 axis, thereby improving oxidative defenses, attenuating oxidative stress, and inhibiting ferroptosis in the gastrocnemius of DMA mice.

**Figure 5:**
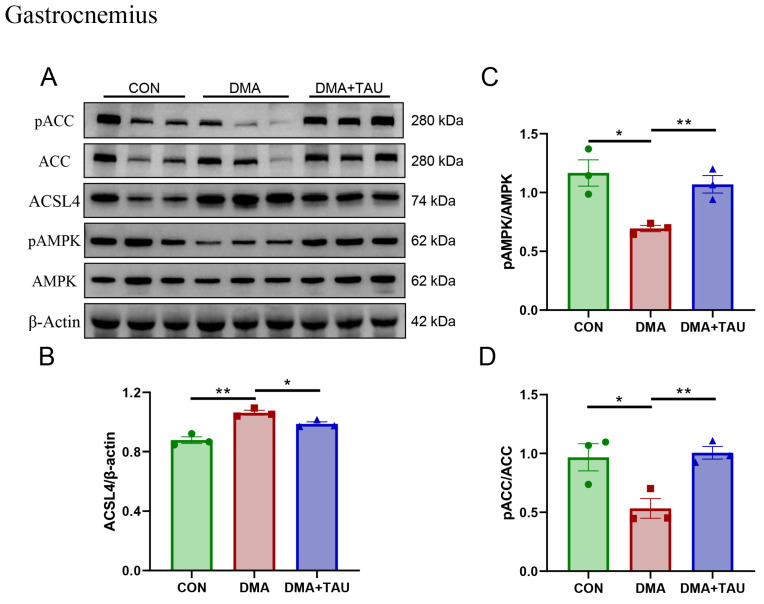
Taurine regulates glycerolipid metabolism associated with the AMPK-ACC-ACSL4 pathways in the gastrocnemius muscle of DMA mice. (**A**,**B**) Western blot analysis and gray level analysis of ACSL4 expression (biological replicates, *n* = 3) showed increased levels of ACSL4 in DMA mice, which taurine reduced. (**A**,**C**,**D**) Phosphorylated AMPK and ACC levels (biological replicates, N = 3) showing significant changes in DMA mice and restoration through taurine intervention. Data are presented as mean ± SEM and analyzed using one-way ANOVA with Tukey’s post hoc test. Statistical significance: * *p* < 0.05, ** *p* < 0.01 vs. DMA.

Concurrently, alterations in glycerolipid metabolism were observed. Elevated levels of sn-glycero-3-phosphocholine and MDA in DMA gastrocnemius muscle suggest heightened phospholipid turnover and oxidative degradation. Taurine intervention attenuated these changes, indicating a protective effect against lipid peroxidation, potentially through modulation of the AMPK-ACC-ACSL4 pathway. Since PUFA-PLs are particularly susceptible to lipid peroxidation, their biosynthesis is critical for ferroptosis susceptibility. This process is regulated by acetyl-CoA carboxylase (ACC), desaturases, and ACSL4, the latter of which catalyzes PUFA-CoA conjugation prior to incorporation into membrane phospholipids [40]. We found that DMA mice exhibited significant upregulation of ACSL4 and reduced phosphorylation of AMPK and ACC, reflecting a dysregulated lipid metabolism that favors PUFA-PL accumulation and ferroptosis (Figure 5A–D).

Importantly, taurine supplementation reversed these pathological changes. It increased the phosphorylation of AMPK and ACC while decreasing ACSL4 expression, suggesting a regulatory role in restoring lipid metabolism and inhibiting ferroptosis. The metabolomics and molecular findings support the conclusion that taurine exerts its protective effects in DMA through coordinated modulation of glutathione and lipid metabolic pathways. This dual-targeted mechanism is further supported by pathway enrichment analyses (Figure S3 and Table S4), reinforcing the role of glutathione and glycerolipid metabolism as key ferroptosis-related processes modulated by taurine treatment.

### 3.5. Taurine Restores the Ferroptosis-Impaired Myogenic Differentiation Potential of C2C12 Myotubes

**Figure 6:**
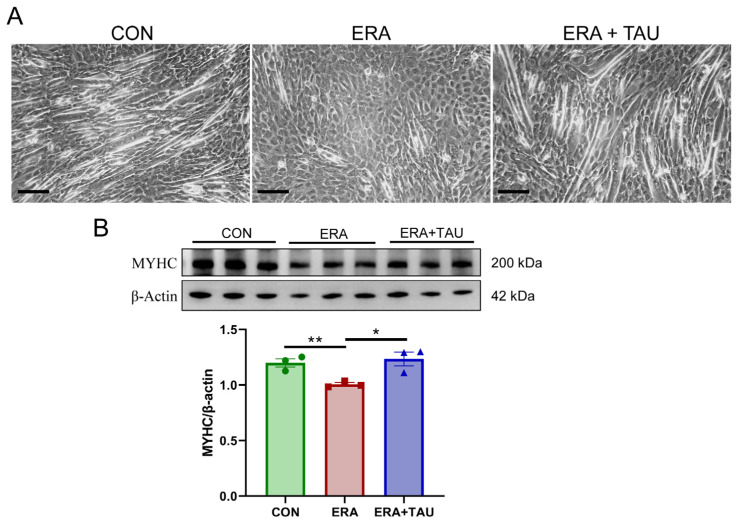
Taurine restores myogenic differentiation potential in ERA-impaired C2C12 myotubes. (**A**) Representative morphological images of myotubes treated with erastin (ERA, 2 μM) alone or in combination with taurine (TAU, 5 mM), showing the protective effect of taurine on myogenic differentiation. Scale bar: 200 μm. Total magnification: 100×. (**B**) Western blot analysis and gray value analysis of MYHC expression showing that taurine significantly restores myogenic differentiation potential (biological replicates, *n* = 3). Data are presented as mean ± SEM and analyzed using one-way ANOVA with Tukey’s post hoc test. Statistical significance: * *p* < 0.05, ** *p* < 0.01 vs. DMA.

**Figure 7:**
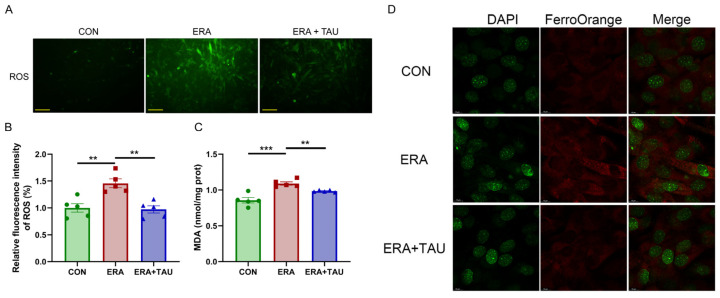
Taurine attenuates ERA-induced oxidative stress in C2C12 myotubes. (**A**,**B**) Fluorescence microscopy images (**A**) and quantification (**B**) of ROS levels in myotubes treated with ERA (2 μM) alone or in combination with TAU (5 mM), (Biological replicates, *n* = 5), demonstrating the ability of taurine to reduce oxidative stress. Scale bar: 200 μm. Total magnification: 100×. (**C**) Quantification of MDA levels in myotubes in different treatment groups, indicating the role of taurine in attenuating lipid peroxidation. (Biological replicates, *n* = 5). (**D**) Confocal fluorescence microscopy images of Fe^2+^ staining illustrating iron accumulation in ERA-treated myotubes and its reduction by taurine. Scale bar: 10 μm. Total magnification: 630×. Data are presented as mean ± SEM and analyzed by using one-way ANOVA with Tukey’s post hoc test. Statistical significance: ** *p* < 0.01, *** *p* < 0.001 vs. DMA.

To explore the mechanistic role of taurine in counteracting ferroptosis-related muscle dysfunction, we investigated the protective effects of taurine on myogenic differentiation impaired by erastin (ERA, the classic ferroptosis inducer) in vitro. ERA was administered in combination with taurine as an intervention. ERA treatment markedly impaired myotube formation and reduced expression of myosin heavy chain (MYHC), indicating impaired myogenic differentiation potential (Figure 6). These effects were accompanied by increased intracellular ROS, MDA, and Fe^2+^ levels, reflecting oxidative and ferroptotic stress (Figure 7).

While these cellular experiments do not replicate the full complexity of disuse muscle atrophy in vivo, they provide complementary mechanistic insights into how ferroptosis disrupts myogenic capacity and how taurine may intervene. Taurine co-treatment reversed ERA-induced myotube damage, reduced oxidative stress markers, and restored MYHC expression.

### 3.6. Taurine Regulates the xCT-GSH-GPX4 and AMPK-ACC-ACSL4 Pathways in Ferroptosis-Injured Myotubes

**Figure 8:**
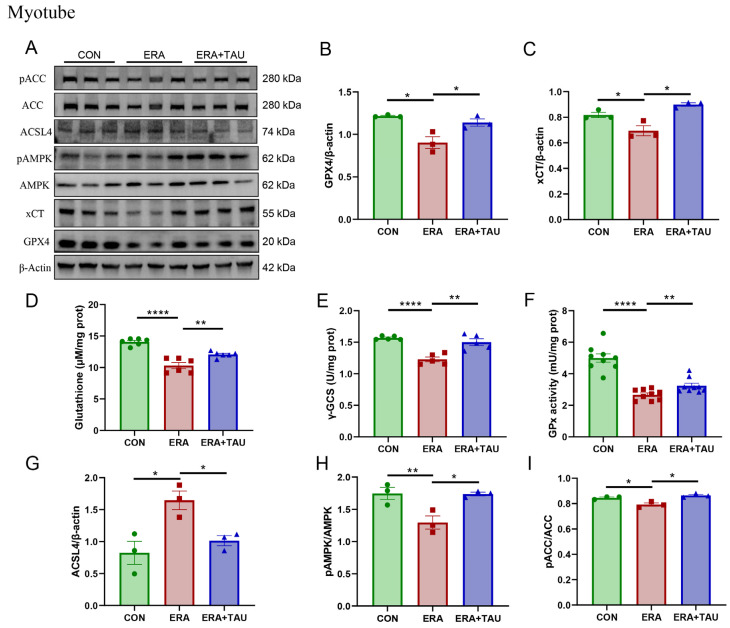
Taurine restores xCT-GSH-GPX4 and AMPK-ACC-ACSL4 pathways in ferroptosis-impaired myotubes. (**A**–**C**) Western blot analysis and gray value analysis of GPX4 and xCT expression in ERA-treated myotubes showing significant restoration due to taurine intervention (biological replicates, N = 3). (**D**) GSH levels (biological replicates, N = 6), (**E**) γ-GCS enzymatic activity (biological replicates, N = 5), highlighting the effect of taurine on GSH biosynthesis. (**F**) GPx enzymatic activity between groups (biological replicates, N = 9), indicating the role of taurine in enhancing antioxidant defenses and the ability of taurine to restore glutathione homeostasis. (**A**,**G**–**I**) Western blot analysis and gray value analysis of ACSL4, phosphorylated AMPK, and ACC, demonstrating taurine-mediated modulation of AMPK-ACC signaling and down-regulation of ACSL4 expression (biological replicates, N = 3). Data are presented as mean ± SEM and analyzed using one-way ANOVA with Tukey’s post hoc test. Statistical significance: * *p* < 0.05, ** *p* < 0.01, **** *p* < 0.0001 vs. DMA.

We further analyzed the effects of taurine on the xCT-GSH-GPX4 and AMPK-ACC-ACSL4 pathways in vitro using C2C12 myotubes treated with ERA. ERA significantly reduced the expression of xCT and GPX4 (Figure 8A–C), the critical regulators of ferroptosis defense axis, along with marked decreases in GSH levels, γ-glutamyl cysteine synthetase (γ-GCS) activity, and GPx activity (Figure 8D–F), reflecting impaired GSH synthesis and antioxidant capacity.

These findings are consistent with the known mechanism of ERA, where xCT inhibition blocks cystine uptake, depleting intracellular GSH and impairing its utilization. Taurine intervention effectively reversed these changes by upregulating xCT and GPX4 expression and elevating γ-GCS and GPx activity, thereby accelerating the rate-limiting step of GSH biosynthesis, improving oxidative defense and inhibiting ferroptosis (Figure 8A–F).

In addition, the AMPK-ACC-ACSL4 lipid metabolic axis was examined. ERA treatment suppressed AMPK and ACC phosphorylation while markedly upregulating ACSL4 expression, indicating increased PUFA synthesis and ferroptotic susceptibility. Taurine supplementation effectively counteracted these changes, as evidenced by reduced ACSL4 expression and increased AMPK and ACC phosphorylation (Figure 8A,B,H–J).

## 4. Discussion

**Figure 9:**
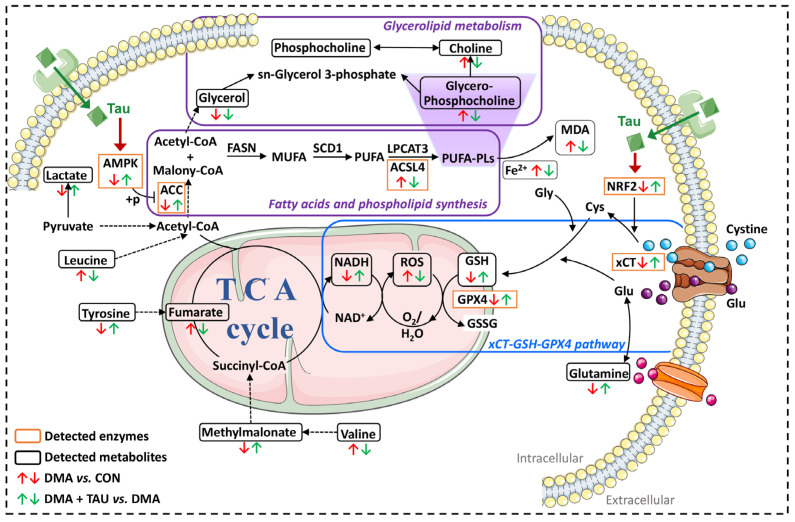
Proposed mechanism by which taurine ameliorates DMA via inhibition of ferroptosis. DMA-induced gastrocnemius atrophy is associated with ferroptotic features, including iron accumulation, GSH depletion, increased MDA and ROS production, and dysregulation of glycerolipid metabolism. Taurine supplementation alleviates these pathological changes by restoring intracellular GSH, reducing iron and MDA accumulation, and mitigating oxidative stress. Specifically, regulation of taurine may be associated with two key protective pathways: (1) the xCT-GSH-GPX4 axis, which enhances cystine uptake, glutathione synthesis, and lipid peroxide detoxification; and (2) the AMPK-ACC-ACSL4 signaling cascade, which inhibits PUFA synthesis and phospholipid peroxidation. While taurine’s ability to modulate ferroptosis-associated metabolic signaling is supported by the observed pathway changes, which converge to inhibit ferroptosis and protect against DMA-induced muscle degeneration, its interaction with specific receptors requires further investigation. Abbreviations: AMPK, AMP-activated protein kinase. ACC, acetyl-CoA carboxylase. ACSL4, acyl-CoA synthetase long-chain family member 4. MDA, malondialdehyde. NADH, reduced form of nicotinamide-adenine dinucleotide. ROS, reactive oxygen species. GSH, glutathione. GPX4, glutathione peroxidase 4. xCT (SLC7A11), solute carrier family 7 member 11.

DMA is characterized by progressive loss of muscle mass and function due to reduced limb activity or prolonged immobilization caused by trauma or microgravity [4]. In this study, we observe for the first time that hindlimb suspension-induced DMA exhibits hallmark features of ferroptosis, including iron accumulation, lipid peroxidation, and decreased expression of xCT and GPX4, as well as reduced GPx activity, implicating ferroptosis as a contributing pathological mechanism in DMA. Importantly, the present study showed that taurine supplementation effectively alleviates DMA, inhibits ferroptosis in DMA, and exerts its protective effect by modulating both glutathione and lipid metabolism, possibly via the xCT-GSH-GPX4 and AMPK-ACC-ACSL4 pathways (Figure 9).

In addition to conventional strategies such as exercise rehabilitation, nutritional interventions—particularly those involving amino acids and antioxidants—are gaining attention for their potential to mitigate DMA [41]. Taurine is one of the most abundant free amino acids in mammalian tissues and has demonstrated cytoprotective roles, including antioxidant and anti-inflammatory effects [21]. Notably, taurine levels increase during the first month after birth in rats, yet they decrease in the skeletal muscle of DMA rats [19,42], suggesting its crucial role in maintaining normal skeletal muscle function.

The taurine dosage of 500 mg/kg used in our study was selected based on previous research demonstrating its efficacy in maintaining muscle health, which support the use of this dosage in our study to investigate the effects of taurine on disuse muscle atrophy [34]. In clinical contexts, taurine has been administered to humans at doses ranging from 1 to 6 g/day for conditions such as cardiovascular disease and diabetes [43]. While the direct translation of animal dosages to humans can be challenging, the dosage of 6 g/day in human studies suggests that our selected dose in mice is within a reasonable range when considering interspecies differences in pharmacokinetics. Here, we found that taurine supplementation increased the indexes of gastrocnemius and soleus, as well as the cross-sectional area of gastrocnemius and grip strength in DMA mice. Moreover, taurine supplementation effectively downregulated the expression of MuRF1, a key E3 ubiquitin ligase associated with muscle degradation [36]. These results underscore the therapeutic potential of taurine in preserving muscle mass and function during periods of disuse.

### 4.1. Taurine Inhibits Ferroptosis in DMA

Chronic disuse exacerbates oxidative stress in skeletal muscle, with the accumulation of ROS recognized as a key driver of muscle atrophy [44]. A major consequence of oxidative stress is the accumulation of lipid hydroperoxides (LOOH), which is exacerbated under conditions of reduced activity of GPX4, a critical enzyme responsible for the reduction of LOOH [45]. This mechanism underlies the process of ferroptosis, a distinct, iron-dependent form of regulated cell death driven by lipid peroxidation that has been implicated in several degenerative diseases.

COX2, an enzyme responsible for the oxidation of PUFAs such as arachidonic acid, has also been identified as the key regulatory target in ferroptosis, acting in concert with key antioxidant regulators such as xCT and GPX4 to modulate redox balance and cellular susceptibility to oxidative damage [13,46]. In the present study, we observed increased COX2 staining, downregulated expression of xCT and GPX4, increased iron and MDA levels, GSH deficiency, and GPX4 defects in DMA gastrocnemius muscle, all consistent with the characteristics of ferroptosis, strongly suggesting that ferroptosis occurs in DMA.

Our previous work has shown that taurine possesses anti-ferroptotic properties in myoblasts, primarily through its ability to enhance antioxidant defenses [23]. Here, our results strongly suggest that taurine significantly reverses muscular atrophy and ferroptosis-associated abnormalities in DMA mice. Specifically, taurine reduced iron and MDA accumulation while restoring xCT and GPX4 expression and replenishing GSH levels, highlighting its therapeutic potential in protecting muscle tissue from ferroptotic damage.

Disuse muscle atrophy has classically been attributed to oxidative stress and apoptosis. There is substantial evidence for caspase activation and mitochondrial apoptotic signaling following immobilization or hindlimb suspension, including increased Bax/Bcl-2 ratios and nuclear DNA fragmentation [8,9,47]. These apoptotic responses are particularly pronounced during the early phases of wasting.

However, apoptosis alone does not fully explain the sustained metabolic and structural deterioration observed during chronic muscle unloading. Our results establish ferroptosis as a potential complementary mechanism of cell death in DMA, characterized by iron overload, GSH depletion, and lipid peroxidation, distinct from the caspase-dependent apoptotic cascade. In particular, the ability of taurine to modulate both iron and lipid metabolism suggests its potential utility in targeting this alternative pathway.

Our study identifies ferroptosis as a major contributor to DMA; however, it is important to recognize that multiple cell death pathways, including apoptosis and necroptosis, may operate concurrently or interactively during muscle degeneration. Although apoptosis has been widely implicated in muscle atrophy, our findings highlight a significant role of ferroptosis. Future studies incorporating markers such as cleaved caspase-3 or TUNEL assays are warranted to delineate how ferroptosis and apoptosis may interact or coexist in DMA. Elucidating such pathway crosstalk could reveal synergistic or compensatory mechanisms driving myofiber loss and help to refine targeted therapeutic strategies.

### 4.2. Taurine Modulates the xCT-GSH-GPX4 Pathway to Improve DMA

Investigating the pathophysiological basis of DMA through metabolic profiling provides valuable insight into therapeutic strategies for muscle preservation [48]. Our NMR-based metabolomic analysis revealed that taurine modulates key metabolic perturbations, particularly those affecting glutathione and glycerolipid metabolism in the gastrocnemius muscle of DMA mice.

GSH, a major antioxidant in the thiol-redox system, is the preferred substrate of glutathione GPX4, an enzyme essential for neutralizing lipid peroxides. GSH biosynthesis depends on cystine uptake via the x_c_^−^ system, whose functional subunit xCT transports cystine into cells, where it is reduced to cysteine and used for GSH production [14,49,50]. Adequate GSH availability enables GPX4 to protect cell membranes from ferroptosis-associated lipid peroxidation, establishing the xCT-GSH-GPX4 axis as a central defense mechanism against ferroptosis [37,51,52].

Upstream of this pathway, the transcription factor NRF2 plays a pivotal role in oxidative homeostasis by inducing the expression of antioxidant proteins, including xCT and GPX4 [53]. Considering the importance of the xCT-GSH-GPX4 pathway in counteracting ferroptosis, it is easy to understand that NRF2 plays a critical role in mitigating lipid peroxidation and ferroptosis [53]. Under oxidative stress, NRF2 is translocated to the nucleus after dissociation from KEAP1 and binds to antioxidant response elements (AREs) to initiate transcription of cytoprotective genes [54]. Notably, NRF2 activation has been shown to attenuate muscle atrophy under various conditions, including aging [55], dexamethasone [56], diabetes [57], and chronic kidney disease [58].

In this study, DMA was associated with significant down-regulation of NRF2, xCT, and GPX4 expression, decreased enzymatic activities of GPx and γ-GCS, and reduced GSH content, which are hallmarks of ferroptosis-driven oxidative damage. Taurine supplementation effectively restored the above abnormalities and NRF2 signaling, and it regulated the xCT-GSH-GPX4 axis. As a result, taurine improved the glutathione metabolic pathway by regulating GSH biosynthesis and utilization, enhancing antioxidant defenses, and inhibiting ferroptosis, ultimately ameliorating DMA.

### 4.3. Taurine Regulates the AMPK-ACC-ACSL4 Pathway to Improve DMA

Phospholipid peroxidation is a key driver of ferroptosis, particularly through the oxidation of PUFA-PLs [15]. Before incorporating into cell membrane phospholipids, fatty acids must first be converted to fatty acyl-CoA, a process catalyzed by the acyl-CoA synthase (ACSL) enzyme family. Among these, ACSL4 preferentially catalyzes PUFA, whereas ACSL3 favors monounsaturated fatty acids (MUFAs) [16,59,60]. Increased ACSL4 activity increases cellular PUFA-PL content, thereby increasing susceptibility to lipid peroxidation and ferroptosis [16,61]. In addition, pharmacological inhibition of ACSL4 has been shown to effectively suppress ferroptosis and attenuate acute organ injury, highlighting its therapeutic relevance [62].

In our study, both DMA gastrocnemius and ERA-treated C2C12 myotubes exhibited significant upregulation of ACSL4 and MDA levels, suggesting an increase in PUFA biosynthesis and enhanced ferroptosis susceptibility. Taurine supplementation significantly downregulated ACSL4 expression in vivo and in vitro, suggesting its role in reducing PUFA accumulation and mitigating lipid peroxidation.

Fatty acid biosynthesis is initiated by the conversion of acetyl-CoA to malonyl-CoA, which is catalyzed by ACC [40,63]. AMPK, the cellular energy sensor, directly phosphorylates and inhibits ACC, thereby suppressing fatty acid synthesis and restoring metabolic balance [64,65]. Recent studies suggest that ACC is a critical downstream target of AMPK in defending against ferroptosis, where AMPK activation inhibits PUFA synthesis by phosphorylating ACC, effectively reducing lipid peroxidation and ferroptosis [66].

In both DMA mice and ERA-treated myotubes, we observed reduced phosphorylation of AMPK and ACC, indicating increased fatty acid biosynthesis and increased susceptibility to ferroptosis. Taurine effectively regulated AMPK and ACC phosphorylation, thereby inhibiting PUFAs biosynthesis and attenuating lipid peroxidation. This was further supported by taurine-induced downregulation of ACSL4, reinforcing the regulatory role of taurine in lipid metabolism and ferroptosis resistance.

Taken together, our findings provide novel insights into the potential role of ferroptosis in DMA and underscore the therapeutic effects of taurine. We observed consistent alterations in ferroptosis-related markers, including increased lipid peroxidation, reduced GSH levels, and dysregulated expression of GPX4 and ACSL4. These results suggest that both the xCT-GSH-GPX4 and the AMPK-ACC-ACSL4 pathways are involved in DMA pathogenesis; their functional roles related ferroptosis are listed in Table S6. The xCT-GSH-GPX4 axis serves as a core antioxidant defense system to facilitate glutathione synthesis and the detoxification of lipid peroxides [12,13,14]. In contrast, the AMPK-ACC-ACSL4 pathway influences ferroptosis susceptibility by modulating PUFA-PL biosynthesis, providing substrates for lipid peroxidation [15,16]. This dual focus is justified by the integrated nature of oxidative and lipid metabolic processes in ferroptosis. Taurine’s modulation of both pathways suggests a multifaceted mechanism of action, targeting both upstream redox regulation and downstream lipid susceptibility. Future studies using genetic or pharmacological inhibition are necessary to validate the interactions and evaluate their potential therapeutic applications in DMA.

Although this study focused primarily on the role of AMPK signaling in lipid metabolism and ferroptosis regulation, it is important to recognize that AMPK also has a well-established role in promoting glucose uptake by enhancing GLUT4 translocation and increasing insulin sensitivity in skeletal muscle. Although we did not assess glucose uptake or blood glucose levels in this study, it is conceivable that taurine-related AMPK regulation may have additional metabolic effects beyond lipid metabolism. Future studies are warranted to evaluate whether taurine supplementation improves glucose homeostasis or alters muscle glucose utilization in the context of disuse muscle atrophy, thereby furthering our understanding of its systemic metabolic benefits.

### 4.4. Prospects and Limitations of the Regulatory Mechanism of Taurine on DMA

While taurine is widely recognized for its antioxidant capacity, our findings suggest that its protective effects in DMA are not solely attributable to general redox modulation. Ferroptosis inhibition involves specific molecular events, such as the suppression of iron accumulation, the prevention of lipid peroxidation, and the modulation of unsaturated fatty acid synthesis, which extend beyond classical antioxidant functions. In this study, taurine was found to increase NRF2 and its downstream targets (GPX4, xCT), which enhance GSH synthesis and antioxidant defense. Taurine also regulates AMPK, which downregulates ACC phosphorylation and reduces the expression of ACSL4, which limits the biosynthesis of polyunsaturated fatty acids and the availability of lipid peroxide substrates. These dual actions—enhancing antioxidant defenses and modulating lipid metabolism—highlight an anti-ferroptotic effect that is distinct yet synergistic with the classical antioxidant role of taurine. Future mechanistic studies using ferroptosis-specifical inhibitors or genetic knockdown models may further elucidate these effects.

It is important to acknowledge several limitations of our study. First, the lack of spontaneous locomotor activity assessments limits the ability to fully interpret taurine’s impact on overall organismal function. Incorporating such behavioral measures, along with electromyography (EMG), in future studies will enable a more comprehensive evaluation of systemic functional outcomes under conditions of muscle disuse and therapeutic intervention. Second, the absence of a classical ferroptosis inhibitor (e.g., ferrostatin-1) as a positive control weakens the mechanistic evidence implicating ferroptosis as a primary contributor to DMA. Future investigations should include such controls to rigorously validate the role of ferroptosis in this context. Third, our study did not assess the long-term effects of taurine intervention, nor did it address its translational applicability to human populations, particularly considering potential sex- and age-related physiological differences. Future investigations should incorporate both male and female subjects to evaluate sex-specific responses and better inform the clinical relevance of taurine as a therapeutic intervention for DMA. Fourth, while our NMR-based metabolomics approach identified several key metabolites associated with muscle atrophy, we were unable to include LC-MS/MS-based independent validation in the current study due to the insufficient number of remaining samples. We acknowledge the importance of confirming metabolite identification using such techniques. Future work should aim to confirm the identified metabolites using independent methods to further strengthen our conclusions.

Looking ahead, there are several key areas including optimizing taurine dosing regimens, evaluating long-term safety, and characterizing its pharmacokinetics in human populations that warrant further investigation to translate these findings into clinical practice. Additionally, prospective clinical trials are needed in at-risk groups, such as bedridden elderly patients, individuals with prolonged postoperative immobilization, and astronauts experiencing microgravity, to evaluate the efficacy, tolerability, and therapeutic potential of taurine in real-world scenarios of disuse muscle atrophy. Specifically, for patients undergoing prolonged bed rest, taurine supplementation may help attenuate disuse-induced muscle atrophy, thereby supporting faster recovery and rehabilitation. In spaceflight settings, taurine could aid in preserving muscle mass and function under microgravity, offering a feasible countermeasure to muscle deconditioning for astronauts during long-duration missions. Moreover, given the prevalence of age-related sarcopenia, taurine may represent a cost-effective and well-tolerated intervention to mitigate muscle loss in elderly individuals, thereby improving mobility and quality of life. Future clinical trials are warranted to evaluate the efficacy, safety, and optimal dosing strategies of taurine in these diverse populations.

## 5. Conclusions

This study provides the first direct evidence that ferroptosis is one of the potential pathological characteristics of DMA, and it demonstrates that taurine supplementation effectively alleviates DMA by inhibiting ferroptosis. We further elucidated the mechanistic basis of taurine’s protective effects, providing new insights into its therapeutic potential. Specifically, we showed that DMA induced by hindlimb suspension leads to hallmark features of ferroptosis, including iron overload, elevated MDA levels, and disruptions in glutathione and glycerolipid metabolism, as well as reduced activity of the xCT-GSH-GPX4 and AMPK-ACC-ACSL4 pathways. These molecular changes result in increased oxidative stress and impaired lipid homeostasis in skeletal muscle. Taurine supplementation, both in vivo and in vitro, restored redox balance and lipid metabolic function by modulating the xCT-GSH-GPX4 axis and the AMPK-ACC-ACSL4 pathway. This resulted in increased glutathione synthesis and utilization, reduced lipid peroxidation, and ultimately attenuation of DMA through the inhibition of ferroptosis. Collectively, these findings deepen our understanding of the pathogenesis of DMA and provide a compelling rationale for targeting ferroptosis and utilizing nutritional strategies such as taurine supplementation as therapeutic approaches for muscle wasting induced by trauma, microgravity, or prolonged disuse. To enhance the rigor and transparency of our findings, we implemented key methodological improvements, including adequate biological replication, appropriate statistical corrections, and comprehensive metabolomic data reporting with fold changes and q-values. Although our results strongly suggest ferroptosis involvement in DMA, future studies using ferroptosis-specific inhibitors are needed to confirm its causal role.

While our findings suggest that taurine-related AMPK-ACC regulation and the suppression of ACSL4 expression may attenuate PUFA synthesis and reduce ferroptosis susceptibility, this proposed mechanism is based on correlative evidence. Future studies using targeted approaches, such as CRISPR/Cas9-mediated knockout or overexpression of ACSL4, or the application of selective pharmacological inhibitors, are essential to validate the causal role of the AMPK-ACC-ACSL4 axis in ferroptosis regulation during disuse muscle atrophy. Additionally, our study demonstrates the acute beneficial effects of taurine on muscle atrophy. Longitudinal assessments should be further investigated in future studies to evaluate the durability of these effects after treatment cessation, which will provide valuable insights into the long-term therapeutic potential of taurine.

## Data Availability

The raw metabolomics data have been uploaded to the Metabolomics Workbench (https://www.metabolomicsworkbench.org/). The study ID is ST003803.

## Supplementary Materials

The following supporting information can be downloaded at: https://www.mdpi.com/article/10.3390/antiox14070847/s1, Figure S1: Representative 1D ^1^H-NMR spectra of aqueous metabolites extracted from mouse gastrocnemius muscle; Figure S2: 2D ^1^H-^13^C HSQC spectra of aqueous metabolites extracted from mouse gastrocnemius muscle; Figure S3: Metabolic pathway analysis of DMA vs. CON and DMA + TAU vs. DMA comparisons; Table S1: NMR resonance assignments of aqueous metabolites extracted from mouse gastrocnemius muscle; Table S2: One-way ANOVA analysis of aqueous metabolites in gastrocnemius muscle across experimental groups; Table S3: Common characteristic metabolites identified from pairwise comparisons of DMA vs. CON and DMA + TAU vs. DMA. Table S4: Significantly altered metabolic pathways identified in the pairwise comparisons of DMA vs. CON and DMA + TAU vs. DMA. Table S5. Significantly altered metabolites across the comparisons. Table S6. Key biomarkers and their roles in ferroptosis.

## Abbreviations

The following abbreviations are used in this manuscript: DMA, Disused muscle atrophy; xCT (SLC7A11), Solute carrier family 7 member 11; GPX4, Glutathione peroxidase 4; GSH, Glutathione; ACSL4, Acyl-CoA synthetase long-chain family member 4; PUFA, Polyunsaturated fatty acid; PUFA-PLs, Polyunsaturated fatty acid phospholipids; MDA, Malondialdehyde; GPx, Glutathione peroxidase; NADPH, Nicotinamide adenine dinucleotide phosphate; ROS, Reactive oxygen species; PCA, Principal component analysis; PLS-DA, Partial least squares discriminant analysis; VIP, Variable importance in projection; PIV, Pathway impact value; NADH, Nicotinamide adenine dinucleotide; MuRF1, Muscle RING finger 1; COX2, Cyclooxygenase 2; MYHC, Myosin heavy chain; AMPK, Adenosine monophosphate-activated protein kinase; ACC, Acetyl-CoA carboxylase.
