# Supplementary material for: Taurine Attenuates Disuse Muscle Atrophy Through Modulation of the xCT-GSH-GPX4 and AMPK-ACC-ACSL4 Pathways
Source: Antioxidants (Basel). 2025 Jul 10;14(7):847. doi: 10.3390/antiox14070847 (PMC12292366; doi:10.3390/antiox14070847)
Supplement: Supplementary file 1 [file antioxidants-14-00847-s001.zip › antioxidants-3675428-supplementary.pdf]

Supplementary Materials

Title: Taurine Attenuates Disuse Muscle Atrophy through Modulation of the xCT-GSH-GPX4 and AMPK-ACC-ACSL4 Pathways

CONTENT

Figure S1. Representative 1D ^{1}H-NMR spectra of aqueous metabolites extracted from mouse gastrocnemius muscle.	p1
Figure S2. The 2D ^{1}H-^{13}C HSQC spectra of aqueous metabolites extracted from mouse gastrocnemius muscle.	p2
Figure S3. Metabolic pathway analysis of the DMA *vs.* CON and DMA+TAU *vs.* DMA comparisons.	p3
Table S1. NMR resonance assignments of aqueous metabolites extracted from mouse gastrocnemius muscle.	p4
Table S2. One-way ANOVA analysis of aqueous metabolites in mouse gastrocnemius muscle between experimental groups.	p6
Table S3. Common characteristic metabolites identified from pairwise comparisons of DMA *vs.* CON and DMA+TAU *vs.* DMA.	p8
Table S4. Significantly altered metabolic pathways and related metabolites identified in the pairwise comparisons of DMA *vs.* CON and DMA+TAU *vs.* DMA	p9
Table S5. Significantly altered metabolites across the comparisons	p10
Table S6. Key Biomarkers and Their Roles in Ferroptosis	p12

Figures and Tables

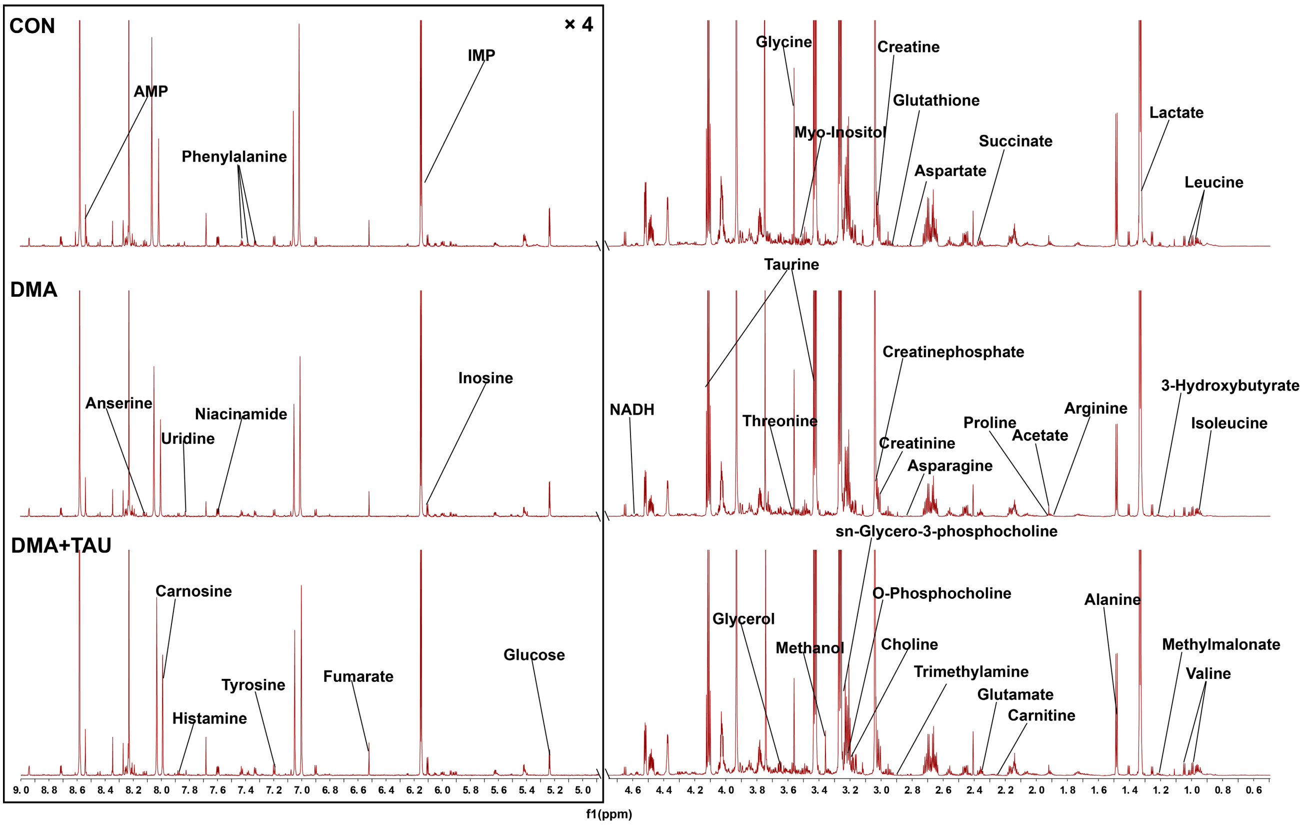

Figure S1. Representative 1D ^{1}H-NMR spectra of aqueous metabolites extracted from mouse gastrocnemius muscle. The spectra were acquired using a Bruker Avance III HD 850 MHz spectrometer at 25 °C. The resonance region of water (4.75-4.85 ppm) has been removed from all the spectra. For clarity, the resonance region from 4.85-9.5 ppm has been magnified fourfold relative to the 0.75-4.75 ppm region. Abbreviations: NADH, nicotinamide adenine dinucleotide; AMP, adenine monophosphate; IMP, ionosine-5'-1-monophosphate.

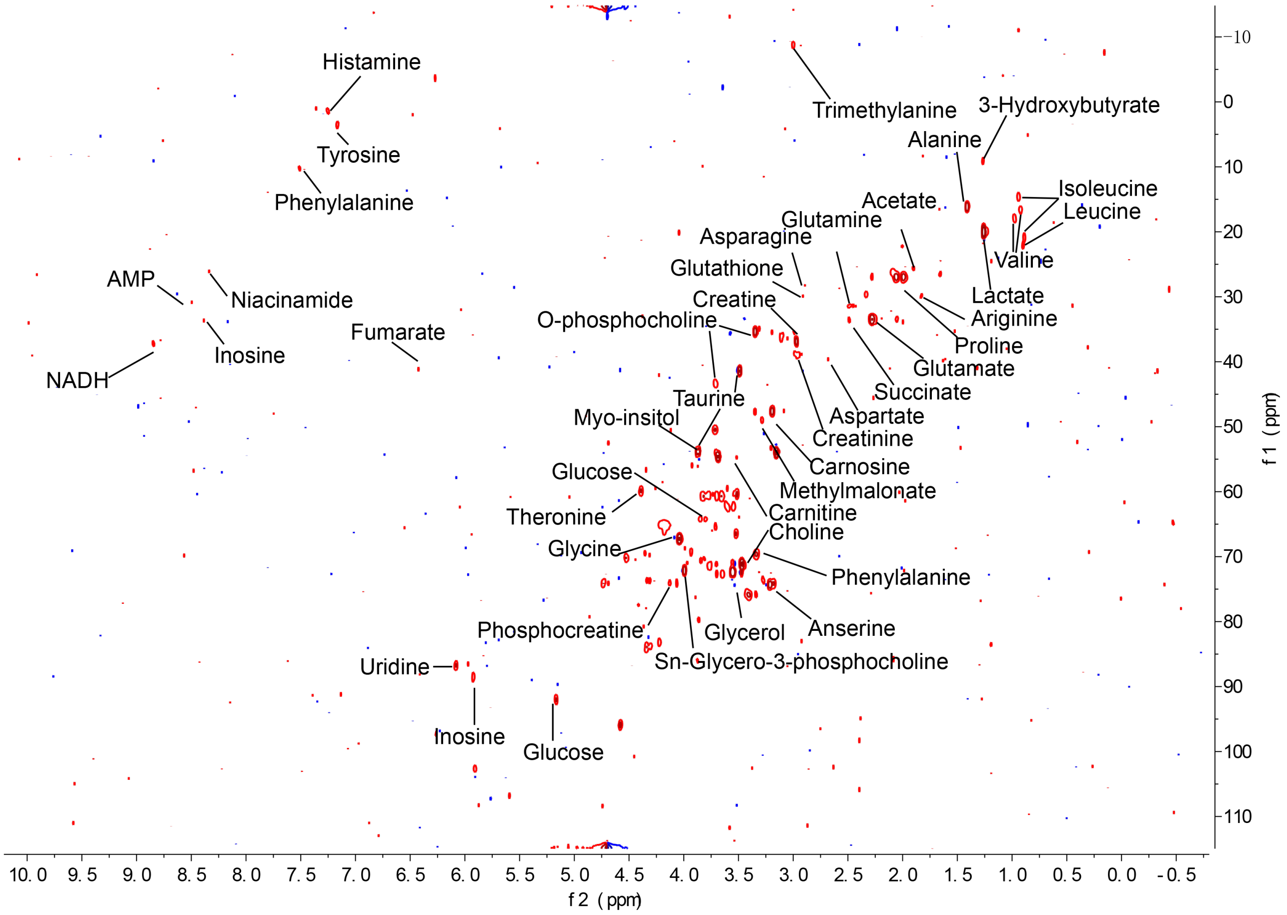

Figure S2. The 2D ^{1}H-^{13}C spectra of aqueous metabolites extracted from mouse gastrocnemius muscle. The spectra were acquired using a Bruker Avance III HD 850 MHz spectrometer at 25 °C.

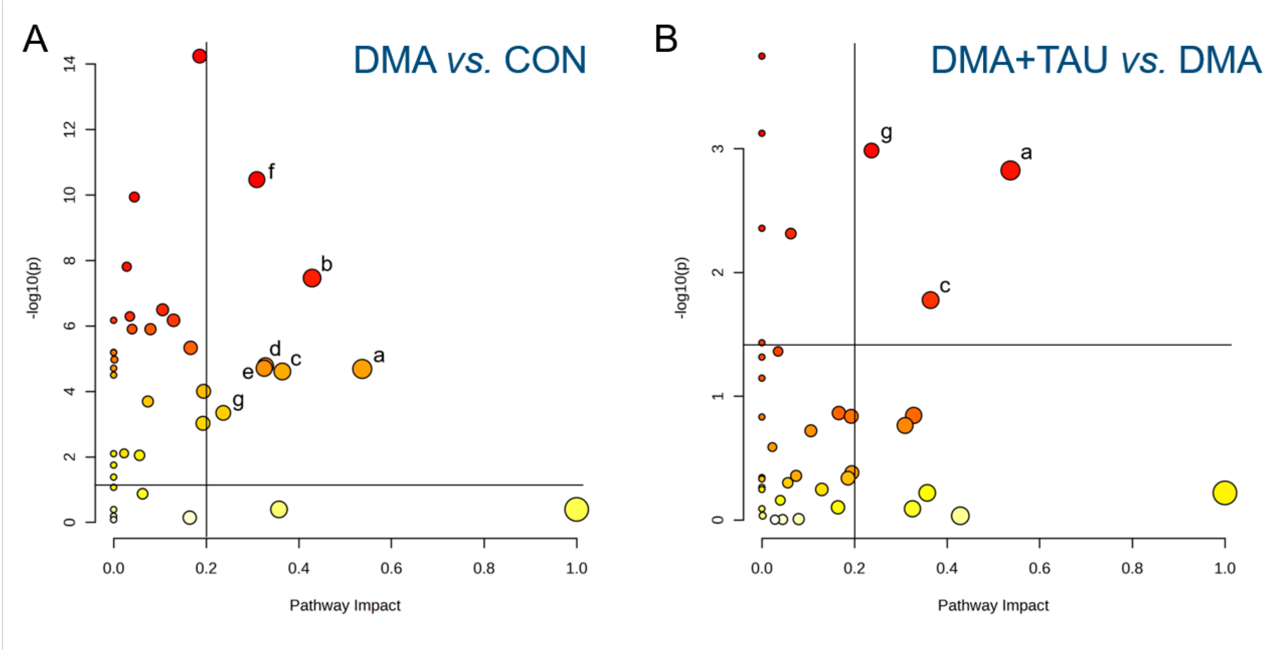

Figure S3. Metabolic pathway analysis of the DMA *vs.* CON and DMA+TAU *vs.* DMA comparisons. The following significantly altered pathways were identified in DMA *vs*. CON (A), including: a. Alanine, aspartate and glutamate metabolism; b. Taurine and hypotaurine metabolism; c. Glutathione metabolism; d. Histidine metabolism; e. Starch and sucrose metabolism; f. Glycine, serine and threonine metabolism; g. Glycerolipid metabolism. Only pathways labeled a, c, and g were identified in DMA+TAU *vs*. DMA (B).

Table S1. NMR resonance assignments of aqueous metabolites extracted from mouse gastrocnemius muscle.

No	Metabolite	δ ^{1}H (ppm) and multiplicity	Moieties
1	Leucine	0.96(d), 0.97(d), 1.69(m), 1.70(m), 1.73(m), 3.73(m)	α-CH_3, α-CH_3, γ-CH, β-CH_2, α-CH
2	Isoleucine	0.94(t), 1.01(d), 1.21(m), 1.42(m), 2.00(m), 3.67(d)	δ-CH_3, γ-CH_3, half γ-CH_2, half γ-CH_2, β-CH, α-CH
3	Valine	0.99(d), 1.05(d), 2.26(m), 3.60(d)	γ-CH_3, γ-CH_3, β-CH, α-CH
4	3-Hydroxybutyrate	1.20(d), 2.30(q), 2.39(q), 4.14(m)	γ-CH_3, β-CH_2, γ-CH
5	Methylmalonate	1.25(d), 3.15(q)	CH_3, CH,
6	Lactate	1.33(d), 4.11(q)	β-CH_3, α-CH
7	Alanine	1.47(d), 3.78(q)	β-CH_3, α-CH
8	Arginine	1.69(m), 1.91(m), 3.24(t)	α-CH_2, β-CH_2, N-CH_2, N-CH
9	Acetate	1.91(s)	CH_3
10	Proline	1.99(m)	γ-CH_2
11	Glutamate	2.08(m), 2.12(m), 2.34(m), 2.37(m), 3.75(m)	half β-CH_2, half β-CH_2, half γ-CH_2, half γ-CH_2, α-CH
12	Glutamine	2.13(m), 2.45(m), 3.77(t)	γ-CH_2, β-CH_2, α-CH
13	Carnosine	7.99(s), 7.05(s),3.24(m)	N-CH-N, N-CH, CH_2
14	Anserine	8.08(s), 7.07(s), 3.2(m), 3.12(q), 2.67(m)	N-CH, N-CH, CH_2, CH_2, CH_2
15	Succinate	2.41(s)	CH
16	Aspartate	2.68(dd), 2.81(dd), 3.90(dd)	β-CH_2, α-CH
17	Asparagine	2.84(dd), 2.94(dd), 4.00(dd)	half β-CH, half β-CH, α-CH
18	Trimethylamine	2.88(s)	CH_3
19	Glutathione	2.15(m), 2.55(m), 2.96(m), 3.77(m), 4.56(m)	β-CH_2, γ-CH_2, CH_2-SH, α-CH&CH_2-NH, CH-NH
20	Creatinine	3.02(s), 4.06(s)	N-CH3, α-CH_2
21	Creatine	3.04(s), 3.93(s)	N-CH3, α-CH_2
22	Creatine phosphate	3.05(s), 4.05(s)	N-CH_3, CH_2
23	Choline	3.20(s), 3.50(dd), 4.03(t)	N-（CH_3）$_3$, N-CH_2, CH_2OH

24	O-Phosphocholine	3.22(s), 3.60(t), 4.18(m)	N-(CH3)3, N-CH2, CH_2OH
25	Sn-Glycero-3-phosphocholine	3.23(s), 3.60(dd), 3.68(dd), 3.87(m), 3.94(m), 4.33(m)	N-（CH_3）$_3$, half $^1CH_2$, $^2CH_2$, half $^1CH_2$, half $^3CH_2$, half $^3CH_2$, $^1CH_2$
26	Carnitine	3.21(s)	CH_3
27	Taurine	3.24(t), 3.41(t)	$^1CH_2$, $^2CH_2$
28	Myo-Inositol	3.28(t), 3.53(dd), 3.63(t), 4.07(t)	^2CH, $^{4,6}CH$, $^{1,3}CH$, ^5CH
29	Glycine	3.57(s)	α-CH_2
30	Threonine	1.31(d), 3.59(d), 4.25(m)	γ-CH_2, β-CH
31	Glycerol	3.57 (dd), 3.66 (dd), 3.79(m)	CH_2, CH_2, CH_2
32	Glucose	Β(3.24(dd), 3.48(t), 3.90(dd)), α(3.54(dd), 3.71(t), 3.72(dd), 3.83(m))	β（H_2, H_3, H_5）, α（H_2, H_3, H_6）
33	Fumarate	6.52(s)	CH
34	Tyrosine	3.05(dd), 3.19(dd), 6.92(d), 7.19(d)	half β-CH_2, half β-CH_2, β-CH, α-CH
35	Phenylalanine	3.12(dd), 3.30(dd), 3.99(dd), 7.33(d), 7.37(t), 7.43(t)	α-CH, half β-CH_2, half β-CH_2, α-CH, β-CH, γ-CH
36	Inosine	8.34(s), 8.24(s), 6.09(d)	CH, CH, CH
37	IMP	8.55(s), 8.22(s)	CH, CH
38	AMP	6.14(d), 8.27(s), 8.58(s)	NH_2, δ-CH, ^2CH
39	NADH	8.44(s)	CH
40	Niacinamide	8.94(d),8.71(dd), 8.24(m)	CH, CH, CH
41	Uridine	7.87(d), 5.92(d), 5.90(d)	N-CH, CH-N, CH
42	Histamine	7.06(s), 7.85(s)	5CH2, 2CH

Note: s: single peak; d: double peaks; t: triple peaks; q: quadruple peak; m: multiple peaks; dd: doublet of doublets.

Table S2. One-way ANOVA analysis of aqueous metabolites in mouse gastrocnemius muscle between experimental groups.

Metabolites	CON	DMA	DMA+TAU	DMA vs. CON	DMA+TAU vs. DMA	F	FDR q
Leucine	0.183±0.006	0.229±0.007	0.212±0.007	↑*	↓	11.279	0.0010
Valine	0.094±0.003	0.122±0.004	0.102±0.004	↑***	↓*	16.579	0.0002
Isoleucine	0.035±0.001	0.047±0.002	0.045±0.001	↑***	↓	21.579	< 0.0001
3-Hydroxybutyrate	0.019±0.003	0.015±0.001	0.023±0.005	↓	↑*	1.355	0.2877
Methylmalonate	0.138±0.003	0.092±0.003	0.103±0.003	↓****	↑	58.362	< 0.0001
Lactate	19.924±0.127	12.401±0.317	14.052±0.403	↓****	↑*	167.745	< 0.0001
Alanine	1.336±0.024	0.987±0.011	0.842±0.028	↓****	↓**	139.542	< 0.0001
Arginine	0.254±0.006	0.165±0.013	0.194±0.005	↓**	↑	26.514	< 0.0001
Acetate	0.053±0.002	0.060±0.003	0.045±0.001	↑	↓**	12.669	0.0006
Proline	0.231±0.006	0.252±0.008	0.198±0.007	↑	↓**	13.969	0.0004
Glutamate	0.235±0.006	0.212±0.004	0.169±0.006	↓*	↓**	38.703	< 0.0001
Glutamine	0.543±0.016	0.321±0.015	0.445±0.023	↓****	↑**	36.229	< 0.0001
Carnosine	0.261±0.007	0.200±0.003	0.222±0.010	↓***	↑	17.133	0.0001
Anserine	0.477±0.006	0.33±0.006	0.342±0.004	↓****	↑	242.415	< 0.0001
Succinate	0.117±0.004	0.123±0.005	0.058±0.004	↑	↓****	61.796	< 0.0001
Aspartate	0.013±0.001	0.014±0.001	0.009±0.001	↑	↓**	9.304	0.0024
Asparagine	0.007±0.001	0.003±0.000	0.004±0.001	↓***	↑	18.020	< 0.0001
Trimethylamine	0.013±0.002	0.005±0.001	0.003±0.001	↓**	↓	15.004	0.0003
Glutathione	0.108±0.004	0.073±0.003	0.124±0.005	↓***	↑****	42.369	< 0.0001
Creatinine	0.421±0.017	0.321±0.014	0.353±0.005	↓***	↑	15.271	0.0002
Creatine	0.515±0.026	0.314±0.026	0.353±0.018	↓***	↑	20.660	< 0.0001
Phosphocreatine	11.001±0.090	8.145±0.110	7.804±0.271	↓****	↓	98.853	< 0.0001
Choline	0.768±0.016	0.383±0.030	0.616±0.020	↓****	↑***	71.358	< 0.0001
O-Phosphocholine	0.698±0.008	0.51±0.009	0.532±0.011	↓****	↑	118.863	< 0.0001

Sn-Glycero-3-phosphocholine	1.036±0.013	1.205±0.061	0.844±0.03	↑*	↓***	20.395	0.0001
Carnitine	0.001±0.000	0.002±0.000	0.001±0.000	↑*	↓*	13.311	0.0005
Taurine	10.728±0.103	8.408±0.187	8.948±0.102	↓****	↑	78.505	< 0.0001
myo-Inositol	0.044±0.001	0.026±0.002	0.032±0.001	↓****	↑*	45.193	< 0.0001
Glycine	0.659±0.015	0.351±0.039	0.442±0.015	↓***	↑	38.519	< 0.0001
Threonine	0.086±0.005	0.068±0.005	0.055±0.007	↓**	↓*	6.273	0.0073
Glycerol	0.294±0.007	0.262±0.008	0.213±0.007	↓*	↓**	32.899	< 0.0001
Glucose	0.102±0.003	0.067±0.003	0.045±0.005	↓****	↓**	69.026	< 0.0001
Fumarate	0.020±0.001	0.025±0.001	0.02±0.002	↑	↓	4.315	0.0331
Tyrosine	0.033±0.001	0.023±0.002	0.029±0.001	↓*	↑	12.241	0.0007
Phenylalanine	0.021±0.000	0.027±0.002	0.022±0.001	↑*	↓	9.419	0.0022
Inosine	0.029±0.000	0.039±0.004	0.039±0.002	↑	↓	5.239	0.0188
IMP	0.842±0.008	0.67±0.013	0.633±0.005	↓****	↓	151.494	< 0.0001
AMP	0.84±0.008	0.642±0.010	0.623±0.004	↓****	↓	244.921	< 0.0001
NADH	0.039±0.006	0.026±0.001	0.029±0.001	↓*	↑*	13.435	0.0003
Niacinamide	0.047±0.001	0.035±0.001	0.036±0.001	↓****	↓	78.043	< 0.0001
Uridine	0.007±0.000	0.005±0.000	0.006±0.000	↓***	↑	11.842	0.0008
Histamine	0.011±0.001	0.008±0.000	0.008±0.000	↓****	↑	26.291	< 0.0001

Note: This table summarizes the statistical analysis of metabolite concentrations in the gastrocnemius muscle of CON, DMA, and DMA+TAU groups. One-way ANOVA with Benjamini-Hochberg correction was used to determine significant changes in metabolite levels between groups. DMA *vs.* CON: Metabolite changes in DMA mice compared to control. DMA+TAU *vs.* DMA: Metabolite changes in taurine-treated DMA mice compared to untreated DMA mice. ↑: Significant increase, ↓: Significant decrease. FDR q-value: Adjusted p-value using the Benjamini-Hochberg method to control for false discovery rate. Data are expressed as mean ± SEM. Statistical significance is indicated as follows: * FDR $q < 0.05$, **FDR $q < 0.01$, ***FDR $q < 0.001$, ****FDR $q < 0.0001$.

Table S3. Common characteristic metabolites identified from pairwise comparisons of DMA vs. CON and DMA+TAU vs. DMA.

Metabolite	DMA *vs.* CON	DMA+TAU *vs.* DMA
Glycerol	↓*	↓**
Alanine	↓****	↓**
Glutamine	↓****	↑**
Threonine	↓**	↓*
NADH	↓*	↑*

Note: This table shows the common characteristic metabolites in both the DMA *vs*. CON and DMA+TAU *vs*. DMA comparisons. The relative concentrations of these metabolites were significantly modulated in the gastrocnemius muscle of DMA mice and subsequently restored by taurine intervention. ↓ indicates a significant decrease, while ↑ indicates an increase compared to the respective control. Statistical significance: *$p < 0.05$, **$p < 0.01$, ****$p < 0.0001$.

Table S4. Significantly altered metabolic pathways and related metabolites identified in the pairwise comparisons of DMA *vs.* CON and DMA+TAU *vs.* DMA

Metabolic pathway	PIV	DMA *vs*. CON		DMA+TAU *vs*. DMA	
		Hits	-lg *p*	Hits	-lg *p*
a. Alanine, aspartate and glutamate metabolism	0.54	7/28	4.69	7/28	2.83
b. Taurine and hypotaurine metabolism	0.43	1/8	7.47	—	—
c. Glutathione metabolism	0.36	3/28	4.61	3/28	1.78
d. Histidine metabolism	0.33	5/16	4.78	—	—
e. Starch and sucrose metabolism	0.33	1/15	4.71	—	—
f. Glycine, serine and threonine metabolism	0.31	4/34	10.47	—	—
g. Glycerolipid metabolism	0.24	1/16	3.35	1/16	2.99

Note: This table summarizes the metabolic pathways significantly altered in DMA and their modulation by taurine treatment. Hits represent the number of altered metabolites within each pathway. -lg *p* denotes the statistical significance of pathway enrichment.

Table S5. Significantly altered metabolites across the comparisons

Metabolite	DMA *vs.* CON		DMA+TAU *vs.* DMA		FDR q	Associated metabolic pathways
		∣Log_2FC∣		∣Log_2FC∣		
Valine	↑***	0.0531	↓*	0.0045	0.0002	Valine, leucine and isoleucine metabolism
Lactate	↓****	0.1628	↑*	0.0023	<0.0001	Glycolysis / Gluconeogenesis, Pyruvate metabolism
Alanine	↓****	0.1047	↓**	0.0530	<0.0001	Alanine, aspartate and glutamate metabolism
Glutamate	↓*	0.0449	↓**	0.0633	<0.0001	Alanine, aspartate and glutamate metabolism, Glutathione metabolism, Histidine metabolism
Glutamine	↓****	0.1874	↑**	0.0173	<0.0001	Alanine, aspartate and glutamate metabolism
Glutathione	↓***	0.0934	↑****	0.1397	<0.0001	Glutathione metabolism
Choline	↓****	0.2173	↑***	0.0489	<0.0001	Glycerophospholipid metabolism, Glycine, serine and threonine metabolism
Sn-Glycero-3-phosphocholine	↑*	0.0071	↓***	0.0324	0.0001	Glycerophospholipid metabolism
Carnitine	↑*	0.1184	↓*	0.2808	0.0005	Thermogenesis
myo-Inositol	↓****	0.1786	↑*	0.0188	<0.0001	Inositol phosphate metabolism, Phosphatidylinositol signaling system
Threonine	↓**	0.1055	↓*	0.0841	<0.0001	Glycine, serine and threonine metabolism
Glycerol	↓*	0.0741	↓**	0.0667	<0.0001	Glycerolipid metabolism
Glucose	↓****	0.2305	↓**	0.0152	<0.0001	Starch and sucrose metabolism
NADH	↓*	0.1354	↑*	0.0258	<0.0001	Oxidative phosphorylation, Thermogenesis

Note: This table summarizes the significantly altered metabolites across the comparisons. One-way ANOVA with Benjamini-Hochberg correction was used to determine

significant changes in metabolite levels between groups. DMA vs. CON: Metabolite changes in DMA mice compared to control. DMA+TAU vs. DMA: Metabolite changes in taurine-treated DMA mice compared to untreated DMA mice. ↑: Significant increase, ↓: Significant decrease. Log_2FC: Log_2-foldchange. FDR q-value: Adjusted p-value using the Benjamini-Hochberg method to control for false discovery rate. Data are expressed as mean ± SEM. Statistical significance is indicated as follows: * FDR $q < 0.05$, **FDR $q < 0.01$, ***FDR $q < 0.001$, ****FDR $q < 0.0001$.

Table S6. Key biomarkers and their roles in ferroptosis

Biomarker	Full name	Functional Role
xCT (SLC7A11)	Solute carrier family 7 member 11	Cystine/glutamate antiporter, critical for glutathione synthesis and redox homeostasis
GSH	Glutathione	Antioxidant that protects against oxidative stress and redox homeostasis
GPX4	Glutathione peroxidase 4	Reduce lipid hydroperoxides using GSH; central to ferroptosis resistance
AMPK	AMP-activated protein kinase	Inhibits lipid biosynthesis via ACC suppression; modulates ferroptosis via energy and lipid metabolism
ACC	Acetyl-CoA carboxylase	Catalyzes conversion of acetyl-CoA to malonyl-CoA; involved in PUFA synthesis
ACSL4	Acyl-CoA synthetase long-chain family member 4	Promotes PUFA-CoA production; increases lipid peroxidation susceptibility
NRF2	Nuclear factor erythroid 2-related factor 2	Master regulator of antioxidant response genes, including xCT, GPX4 and so on
MDA	Malondialdehyde	Biomarker of lipid peroxidation and oxidative damage
Iron		Required for lipid peroxidation and ferroptosis induction
